# Composition-Dependent SERS Responses of Structurally Related Dyes on Inkjet-Printed Paper Substrates with Gold and Silver Triangular Nanoprisms

**DOI:** 10.1021/acsomega.6c04353

**Published:** 2026-07-20

**Authors:** Lucas M. F. Oliveira, Anerise de Barros, Edison Huertas-Montoya, Gabriel C. Pereira, Diego P. dos Santos, Fernando A. Sigoli, Italo O. Mazali

**Affiliations:** 28132 Universidade Estadual de Campinas, Instituto de Química, Laboratório de Materiais Funcionais, Campinas, São Paulo 13083-970, Brasil

## Abstract

Inkjet-printed paper-based surface-enhanced Raman scattering (SERS) substrates incorporating gold triangular nanoprisms (AuTNPs) and silver triangular nanoprisms (AgTNPs) were prepared to investigate how nanoparticle composition influences the SERS response of structurally related dyes, crystal violet (CV) and malachite green (MG). AuTNPs and AgTNPs were synthesized, formulated into printable inks, and deposited side by side onto hydrophobized chromatography paper. Scanning electron microscopy (SEM) and optical characterization confirmed the preservation of nanoprism morphology after printing, while revealing superior spectral reproducibility and storage stability for AuTNP-based substrates compared to AgTNP-based ones. The SERS spectra of CV and MG exhibited composition-dependent vibrational fingerprints, with AuTNPs providing higher enhancement factors and inducing systematic shifts and relative intensity variations in characteristic Raman bands. Multivariate analysis based on artificial neural networks (ANNs) was applied to binary CV/MG mixtures, yielding mean prediction errors (MSE) of 0.080 for the training set and 0.106 for the validation set, with larger deviations associated with lower-enhancement regions. Overall, the results demonstrate that nanoprism composition plays a critical role in shaping SERS spectral features and that combining signals from different plasmonic nanostructures on a printed paper substrate enhances the spectroscopic discrimination and quantitative analysis of closely related molecular species.

## Graphical abstract

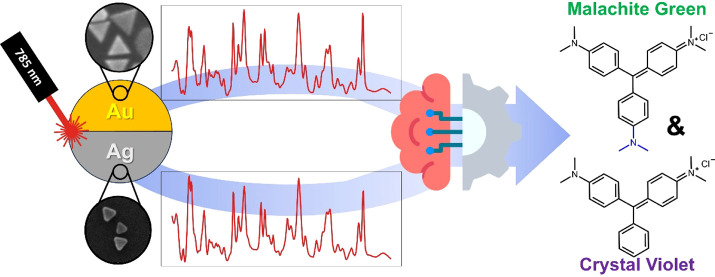

## 1 Introduction

Surface-enhanced Raman spectroscopy (SERS) is a powerful spectroscopic technique that enables the detection of weak Raman signals by amplifying molecular vibrational responses, thereby extending the applicability of Raman spectroscopy to trace-level analysis of environmentally and biologically relevant species.^1^ Signal enhancement is primarily achieved through the interaction of analytes with plasmonic nanostructures, typically composed of gold or silver, either in colloidal form or immobilized on solid substrates.^2,3^ These nanostructures generate highly localized electromagnetic fields upon optical excitation, which strongly amplify Raman scattering. In addition to the dominant electromagnetic mechanism, a chemical enhancement mechanism may also contribute by modifying the polarizability of adsorbed molecules through charge-transfer interactions.^4^ Both mechanisms depend critically on the proximity and orientation of the analyte relative to the nanostructured surface, which can result in changes in band intensities, selection rules, and relative mode activation.^5^ Consequently, SERS spectra provide not only molecular fingerprints but also detailed information on molecule–surface interactions and adsorption geometries.^6^ This sensitivity to nanostructure morphology and surface chemistry has been demonstrated, for example, by Scher et al.,^7^ who showed that molecules such as crystal violet, 4-aminothiophenol, and thiophenol exhibit distinct SERS responses when adsorbed on gold nanostars with different morphologies.

Conventional SERS substrates typically consist of metallic nanoparticles dispersed in colloidal solutions or immobilized on rigid supports such as silicon, quartz, or glass. However, the fabrication of these substrates often relies on complex and costly methods, including nanolithography and multistep surface modification procedures, which may limit reproducibility and scalability.^8^ The increasing availability of compact and portable Raman spectrometers has motivated the exploration of alternative, flexible substrates that enable simpler fabrication and improved adaptability to diverse sampling scenarios.^9^ In this context, paper has emerged as an attractive support for SERS applications due to its low cost, ease of handling, capillary-driven sample transport, and renewable nature.^10,11^ Moreover, the entangled cellulose fiber network in paper can effectively immobilize nanoparticles and promote the formation of three-dimensional plasmonic hot spots, thereby enhancing Raman signal amplification.^12^ For example, by soaking filter paper in salt-induced aggregated Ag/Au nanoparticles, Moram et al.^13^ developed an efficient SERS substrate capable of detecting multiple explosive molecules.

Inkjet printing of metallic nanoparticle inks onto paper has recently gained attention as a versatile approach for the controlled and automated fabrication of SERS substrates. Importantly, this method is compatible with a wide range of nanoparticle morphologies. For instance, Godoy et al.^14^ employed inkjet-printed gold nanospheres for the detection of the pesticide thiram, while Huertas-Montoya et al.^15^ utilized printed gold nanostars for the identification of the psychoactive compound psilocybin. In both cases, standard commercial printers enabled reproducible substrate fabrication and sensitive detection at low analyte concentrations. Joshi et al.^16^ enhanced the stability of silver nanostructures by implementing a print–expose–develop workflow, inspired by silver-halide photography, to fabricate on-demand SERS-active silver nanowire networks, also using a standard desktop inkjet printer. Tay et al.,^17^ in turn, developed paper-based SERS sensors by inkjet-printing a colloidal Au sol onto filter paper for the detection of chemical aerosols, and proposed the use of receiver operating characteristic analysis as an alternative measurand to quantify sensor performance, accounting for the heterogeneous nanoparticle loading inherent to paper substrates. In a subsequent stage, they employed a precision materials printer to deposit controlled, quantifiable amounts of analyte (fentanyl) uniformly across the active sensing region of the paper SERS platform, enabling the preparation of analyte-loaded certified reference materials for field deployment and comparative standardization.

Beyond spherical nanoparticles, anisotropic nanostructures with sharp features are of particular interest for SERS, as they generate intense localized electromagnetic fields at tips and edges. Gold triangular nanoprisms (AuTNPs), characterized by well-defined planar geometry and symmetric tip arrangements, have been shown to provide stronger plasmonic enhancement than many conventional gold nanostructures, including nanospheres, hexagonal nanoplates, nanorods, and bipyramids, with the exception of gold nanostars.^18^ While nanostars often exhibit superior enhancement due to multiple tips, achieving uniform size and shape remains challenging, whereas AuTNPs offer improved morphological consistency. Silver triangular nanoprisms (AgTNPs) also display promising SERS performance and have been applied, for example, to in situ spectroscopic monitoring in heterogeneous catalysis when immobilized on porous membranes,^19^ as well as to pesticide detection when supported on flexible wool fibers.^20^

Given the impact of analyte–surface interactions on the SERS spectrum, this study aimed to investigate whether the composition of nanoprisms could influence the SERS spectra of crystal violet (CV) and malachite green (MG), two commonly used model molecules and potential environmental pollutants due to their application in aquaculture and possible carcinogenicity.^21^ The literature offers numerous examples of SERS substrates for the detection of CV and MG. These include surface-modified silver nanowires embedded into polydimethylsiloxane,^22^ gold nanorods nanoparticles hybridized with poly­(vinyl alcohol) hydrogel,^23^ self-assembled silver nanostar film interfaced with flexible polytetrafluoroethylene filter membrane^24^ and silver nanoparticles-embedded nylon filter membrane.^25^ In the present work, we further examine whether variations in nanoparticle composition lead to measurable differences in vibrational fingerprints and whether the relative proportions of CV and MG in aqueous mixtures can be quantitatively assessed based on their SERS spectra. To enable direct comparison under identical experimental conditions, inkjet printing was employed to deposit AuTNPs and AgTNPs inks side by side on the same hydrophobized paper substrate, providing a controlled platform for investigating composition-dependent SERS responses. Inkjet printing enables controlled tuning of nanoparticle surface coverage, which facilitates direct comparison between nanoprism compositions while also supporting mass-scale production and commercial viability. The hydrophilization of the paper substrate allows aqueous analyte solutions to be drop-cast onto the surface, acting as a preconcentration step and reducing signal variability on paper substrates.^26,27^

## 2 Results and Discussion

### 2.1 Substrate Characterization

SEM micrographs show that the average edge length of AgTNPs is (67 ± 13) nm, while that of AuTNPs is (65 ± 18) nm, as can be seen in Figure 1a and Figure 2a. Additional SEM images for AgTNPs and AuTNPs are included in Figures S2 and S3. Notably, the tips of the AuTNPs appear sharper and more well-defined than those of the AgTNPs, which can be attributed to differences in the synthesis routes employed as well as to the well-documented lower structural stability of silver nanoparticles.^28^ The most intense bands in each ink’s extinction spectrum (Figure 1b and Figure 2 b) correspond to the in-plane dipole resonance of the nanoprisms, which is highly sensitive to changes in nanostructure or variations in the surrounding medium’s properties, such as refractive index during drying.^29^ Accordingly, the band of the AgTNPs ink, centered at 593 nm in the colloid, undergoes a slight shift to 599 nm upon printing (Figure 1c). Meanwhile, the band of the AuTNPs ink, initially at 654 nm in suspension, appears at 590 nm after printing (Figure 2c). Thus, despite differences in composition and size distribution, the printed nanoprisms exhibit a comparable maximum plasmonic response. SEM micrographs of the printed substrates, demonstrating that the morphology remains unchanged upon printing, are provided in the Supporting Information: Figure S4a, b for AgTNPs and AuTNPs.

**Figure 1:**
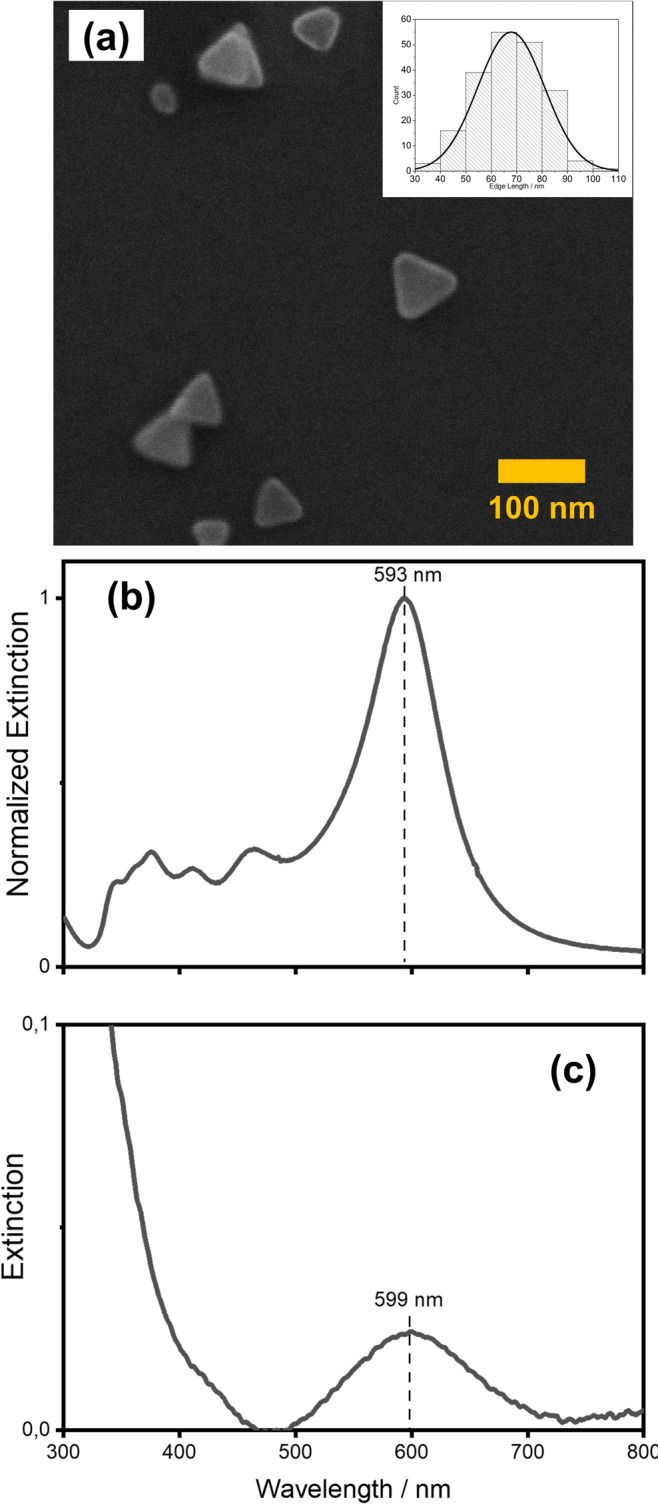
(a) SEM micrograph of silver triangular nanoprisms (AgTNPs); inset shows the corresponding edge-length distribution histogram. (b) Normalized VIS–NIR extinction spectrum of the AgTNP ink, normalized to the maximum intensity band. (c) Baseline-corrected diffuse reflectance VIS–NIR extinction spectrum of the inkjet-printed AgTNP substrate.

**Figure 2:**
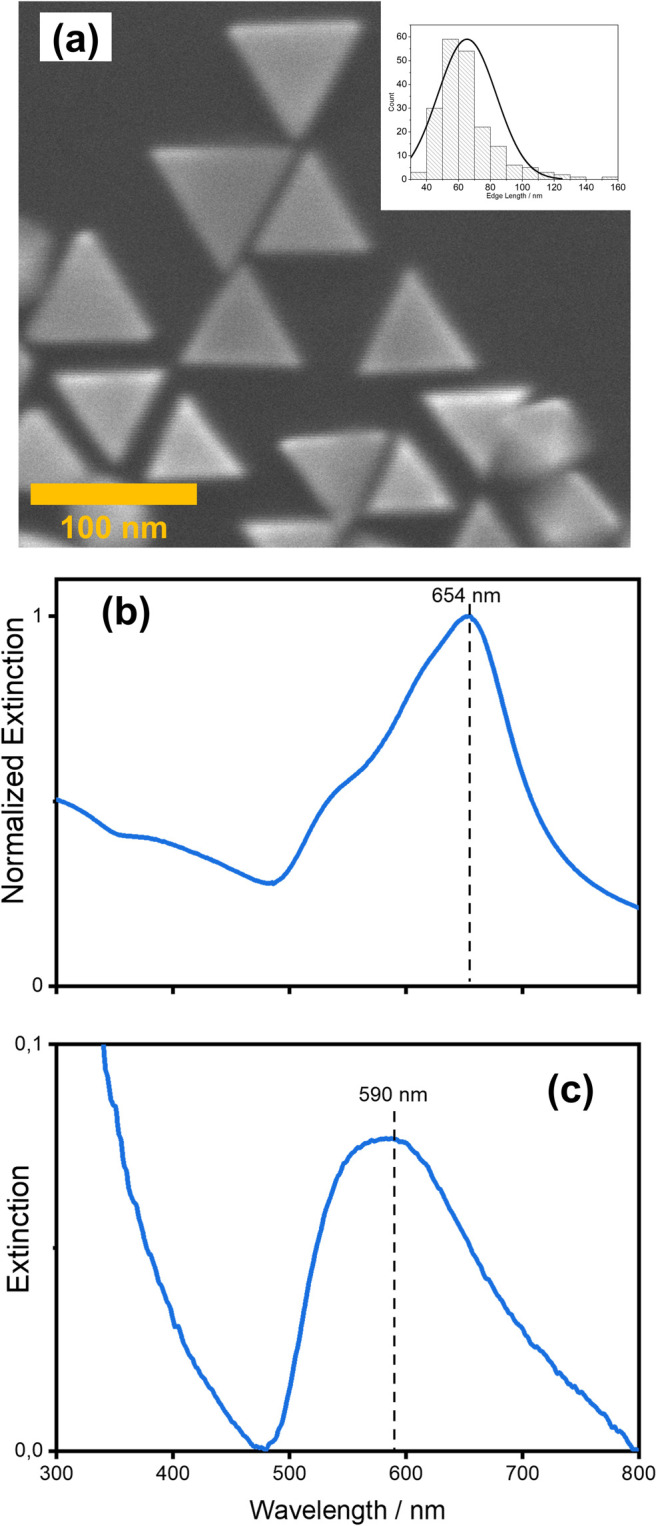
(a) SEM micrograph of gold triangular nanoprisms (AuTNPs); inset shows the corresponding edge-length distribution histogram. (b) Normalized VIS–NIR extinction spectrum of the AuTNP ink, normalized to the maximum intensity band. (c) Baseline-corrected diffuse reflectance VIS–NIR extinction spectrum of the inkjet-printed AuTNP substrate.

The spectroscopic stability of both the nanoprism inks and the corresponding printed substrates was also evaluated. Although no extensive shifts were observed in the extinction bands of the AgTNPs ink over six months of storage under ambient conditions and protected from light, as seen on Figure S5a in the Supporting Information, a “silver mirror” effect was noted inside the storage vial by the ninth month. This colloidal instability may be attributed to the initially low magnitude of the zeta potential (−9.4 ± 0.2) mV (PDI: 0.5), which suggests limited electrostatic stabilization.^30^ Over the same storage period, the extinction spectrum of the AuTNPs ink (initial zeta potential: (−1.7 ± 0.1) mV; (PDI: 0.7) exhibited a gradual blueshift of approximately 50 nm (Figure S5b). In contrast to the inks, the printed substrates showed markedly different stability behaviors. The AgTNPs-printed substrate was unstable under atmospheric conditions, with the extinction band disappearing after one month of storage in air (Figure S5c). Although X-ray photoelectron spectroscopy (XPS) was not performed, the progressive loss of the plasmonic extinction band strongly indicates silver oxidation. This interpretation is further supported by the fact that the band remained detectable for up to three months when the samples were stored in a vacuumed desiccator, Figure S5e. The AuTNPs-printed substrate exhibited superior stability under both storage conditions. When stored in a desiccator, its extinction spectrum remained largely unchanged over the evaluated period (Figure S5f), while under atmospheric exposure the degradation was significantly less pronounced than that observed for the AgTNPs-printed substrate (Figure S5d).

### 2.2 Substrate Performance

The CV SERS spectrum exhibits apparent differences depending on the metal of the nanoprisms it interacts with, as can be seen in Figure 3a. Notably, new bands appear at 1222 cm^–1^, linked to ν_s_(CC_center_C)/δ­(CCC)_breathing_/δ­(CH),^31^ and at 759 cm^–1^, related to ν_s_(CC_center_C)/ν­(CN),^31^ which are not present in CV deposited on functionalized paper. There’s also a rise in the intensity of bands at 938 cm^–1^, associated with ρr (CH_3_)/ν­(CN),^31^ and at 1171 cm^–1^, ν_s_(CC_center_C)/δ­(CCC)_breathing_/ρ_r_(CH_3_).^31^ However, the most notable difference is in the pair of bands linked to benzene ring stretching:^31^ the band at 1584 cm^–1^ shifts to 1581 cm^–1^ for both printed nanoprisms, while the band at 1617 cm^–1^ shifts to 1614 cm^–1^ for AgTNPs-printed and to 1610 cm^–1^ for AuTNPs-printed. Additionally, the intensity ratio between these two bands changes from 1 to 1.5 for AgTNPs-printed and to 2.2 for AuTNPs-printed. Despite these variations, the average SERS and Raman spectra are quite similar, as calculated using Spectral Angle Mapper (SAM):^32^ 66.2% between the two nanoprisms, 68.6% between CV spectra on functionalized paper and on AgTNPs, and 42.8% for AuTNPs. The mathematical basis of SAM involves representing the reference spectra (in this case, the molecule on functionalized paper) and the other spectra as vectors in a space defined by the number of spectral bands, and determining the angle formed between these vectors.^32^ The main spectral features of CV are summarized in Table 1, along with the corresponding features for MG.

**Table 1:** Main Raman and SERS Spectral Features of Crystal Violet (CV) and Malachite Green (MG) as a Function of the Interacting Surface

Crystal violet			
Mode^a^/cm^–1^	On functionalized paper	On AgTNPs-printed	On AuTNPs-printed
ν_s_(CC_center_C) or ν(CN)	-	759	759
ρ_r_(CH_3_) or ν(CN)	938	938	938
ν_s_(CC_center_C) or δ(CCC)_breathing_ or ρ_r_(CH_3_)	1171	1171	1171
ν_s_(CC_center_C) or δ(CCC)_breathing_/δ(CH)	-	1222	1222
ν_s,1_(CC)_ring_	1584	1581	1581
ν_s,2_(CC)_ring_	1617	1614	1610
ν_s,2_(CC)_ring_ /ν_s,1_(CC)_ring_	1	1.5	2.2
	On AgTNPs-printed vs On AuTNPs-printed	On AgTNPs-printed vs On functionalized paper	On AuTNPs-printed vs On functionalized paper
Similarity/%	66.2	68.6	42.8

Malachite green			
Mode^b^/cm^–1^	On functionalized paper	On AgTNPs-printed	On AuTNPs-printed
ν_s_(CC_center_C) or ν(CN)	755	759	759
γ(CH)	1171	-	-
ν(CN)	1376	1376	1364
			1391
ν(CC)_ring_	-	1448	1444
ν_s,1_(CC)_ring_	1588	1588	1588
ν_s,2_(CC)_ring_	1614	1614	1610
ν_s,2_(CC)_ring_ /ν_s,1_(CC)_ring_	3.9	2.9	3.2
	On AgTNPs-printed vs On AuTNPs-printed	On AgTNPs-printed vs On functionalized paper	On AuTNPs-printed vs On functionalized paper
Similarity/%	54.2	79.8	37.2

a Cañamares et al.;^31^

b Liang, F. et al.^33^

**Figure 3:**
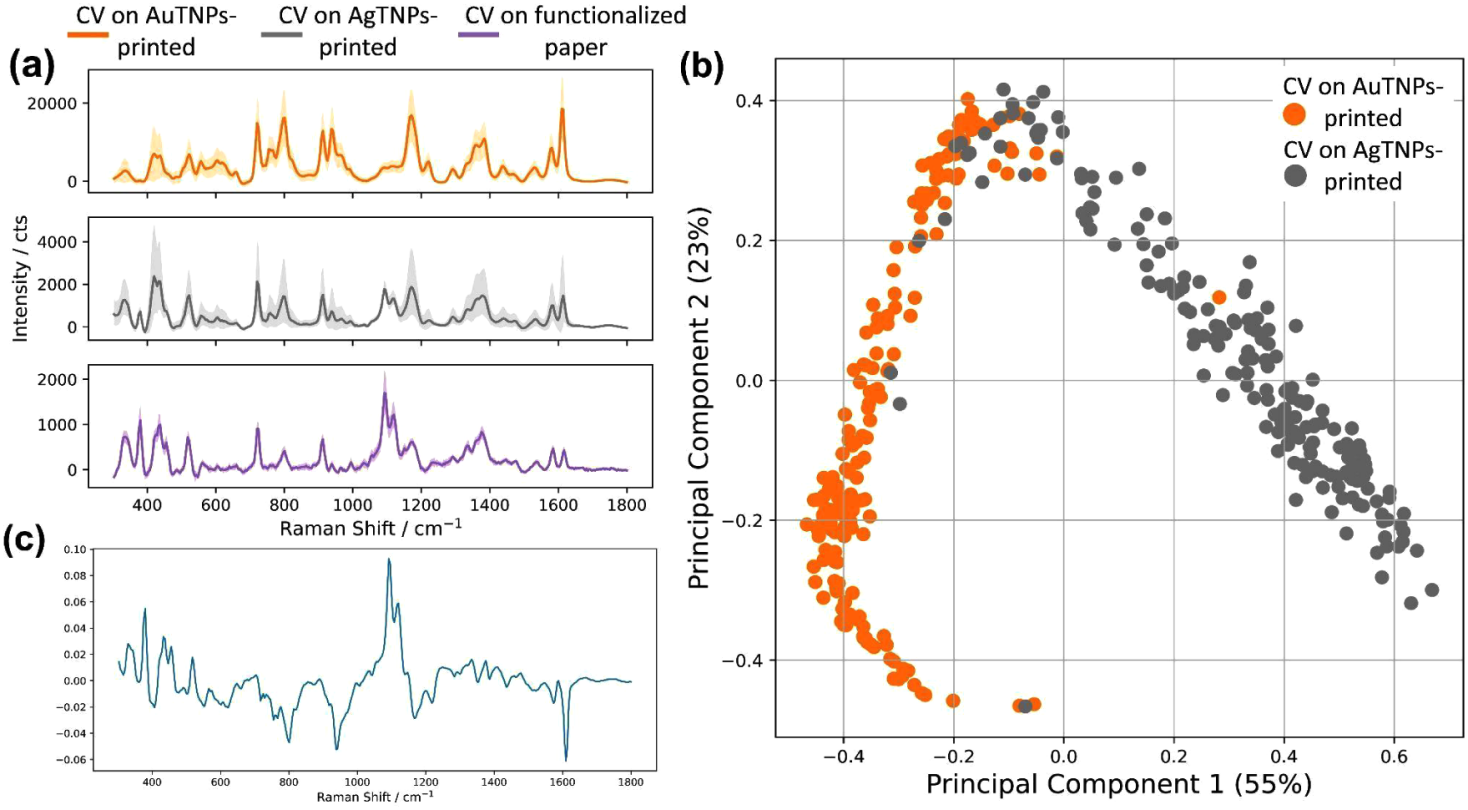
(a) Average Raman and SERS spectra of crystal violet (CV, 10 μmol L^–1^) interacting with functionalized paper, AgTNPs-printed, and AuTNPs-printed; shaded regions represent the corresponding standard deviations. (b) Principal component analysis (PCA) score plot of CV spectra after normalization. (c) Loading plot of the first principal component (PC1).

The SERS spectra of CV obtained on the two types of triangular nanoprisms are sufficiently distinct that principal component analysis (PCA) reveals a clear clustering tendency according to nanoparticle composition (Figure 3b). Interpretation of the loading plot for the first principal component (Figure 3c) indicates that CV-related vibrational bands are primarily responsible for the separation observed for the AuTNPs-printed based spectra, consistent with the spectral differences discussed above. In contrast, the separation of the AgTNPs-printed based spectra is strongly influenced by bands associated with the functionalized paper substrate, particularly in the 300–600 cm^–1^ and 1050–1150 cm^–1^ regions. This occurs because the CV spectra are significantly more intense on AuTNPs-printed compared to AgTNPs-printed, according to the Analytical Enhancement Factor (AEF) calculated based on the second ν_benzene_ band is 3.4 for AgTNPs-printed and 43.5 for AuTNPs-printed, using eq 1. Thus, although the spectra at the printing interface exhibit features from both nanoprisms, AuTNPs-printed dominate, which explains the incomplete spectral separation observed in Figure 3b. Similar data interpretation for MG can be inferred from the data shown in Figure 4a–c.

1 $$\mathrm{AEF}=\frac{I_{\mathrm{SERS}}\times C_{\mathrm{SERS}}}{I_{\mathrm{Raman}}\times C_{\mathrm{Raman}}}$$

whereas I_SERS_ refers to the second ν_benzene_ band of CV respective to the AgTNPs-printed or AuTNPs-printed; I_Raman_ was assumed to be the intensity of the second ν_benzene_ band of CV onto the DDSA-hydrophobized paper; C_Raman_ corresponds to the concentration of the drop casted solution of CV (10 μmol L^–1^); assumed to be equal to C_SERS._

**Figure 4:**
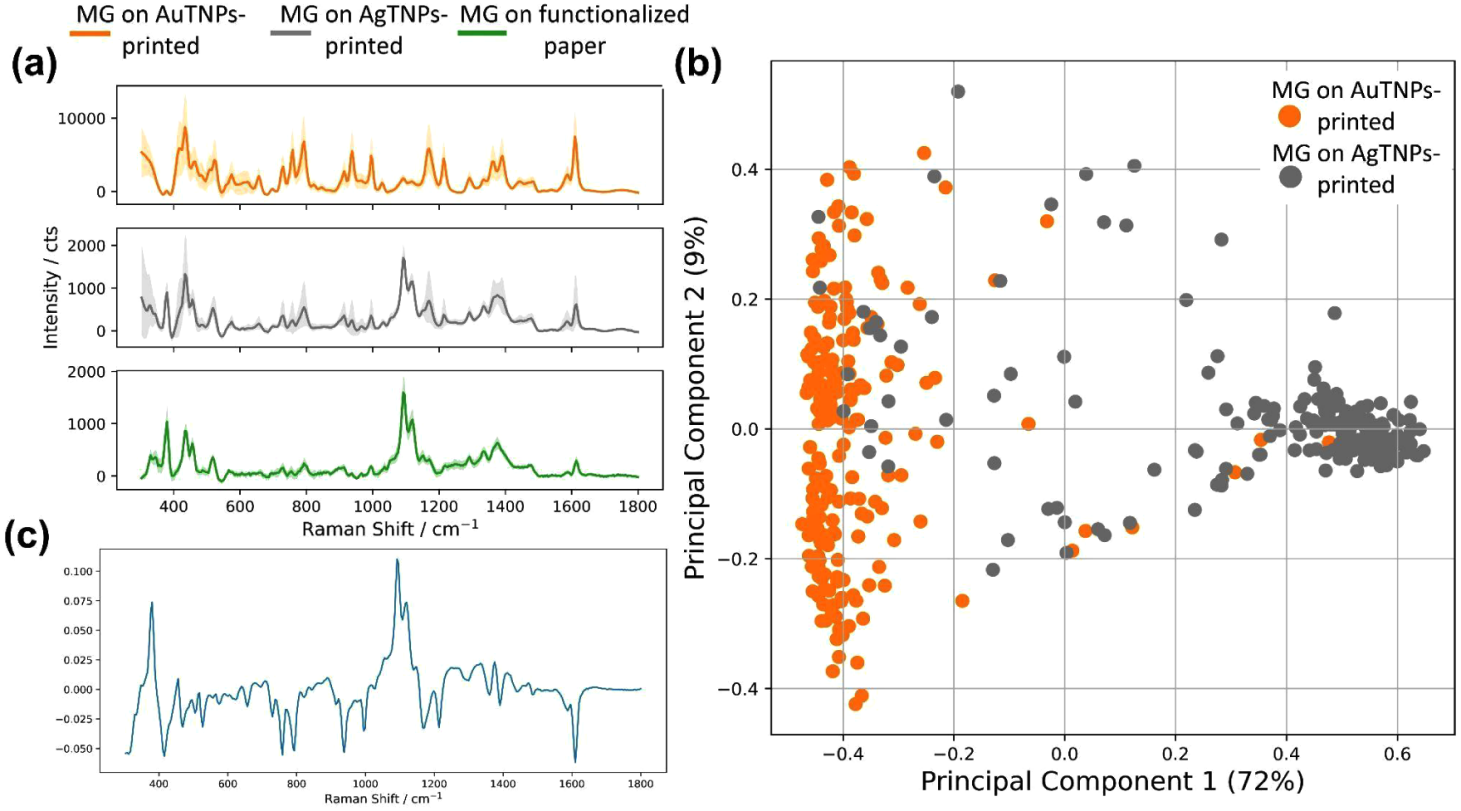
(a) Average Raman and SERS spectra of malachite green (MG, 10 μmol L^–1^) interacting with functionalized paper, AgTNPs-printed, and AuTNPs-printed; shaded regions represent the corresponding standard deviations. (b) Principal component analysis (PCA) score plot of MG spectra after normalization. (c) Loading plot of the first principal component (PC1).

Compared to CV, MG presents an additional dimethylamine group. However, the most notable differences between the spectra in Figure 3a (CV) and Figure 4a (MG) lie in the relative band intensities, particularly in the ratio of the pair of bands associated with benzene-ring stretching. PCA analysis also reveals a clustering trend based on nanoprisms composition (Figure 4b), and interpretation of the loading plot for the first principal component (Figure 4c) reflects the influence of the bands observed in the printed substrate for the separation of MG interacting with AgTNPs-printed, while the MG-related bands drive the separation of AuTNPs-printed spectra. Additionally, a series of SERS spectra from printed substrate regions with varying CV/MG proportions is presented in Figure S6a–e for AgTNPs-printed and Figure S7a–e for AuTNPs-printed.

The spectral differences observed for CV and MG adsorbed on AuTNPs and AgTNPs can be rationalized considering the combined effects of vibrational selection rules, adsorption geometry, molecule–surface electronic interactions, and the intrinsic plasmonic properties of the metallic nanoprisms. For both dyes, the most prominent Raman bands correspond to aromatic ring at 938 and 1171 cm^–1^ are attributed to C–H in plane bending and C–N stretching vibrations, while the bands around 1581 and 1610–1614 cm^–1^ correspond to phenyl ring stretching modes. Similar assignments apply to MG, whose spectra are dominated by aromatic C=C stretching and dimethylamino-related vibrations. The observed shift peak position and relative intensity between AuTNPs (1610 cm^–1^) and AgTNPs (1614 cm^–1^) indicate differences in adsorption orientation and interfacial electronic coupling.^34^ Based on SERS surface rules, vibrational modes with polarizability components perpendicular to the metal surface are preferentially enhanced. Therefore, changes in the relative intensity ratios suggest distinct adsorption geometries on AuTNPs and AgTNPs. The stronger enhancement of specific aromatic stretching modes on AuTNPs suggests a more favorable orientation for coupling between the molecular π-system and the localized plasmonic field. This indicated highly polarizable ring modes with the enhanced electromagnetic field localized at the nanoprism edges tips. The peak shifts also provide evidence of different molecule–surface interactions.^31^

A frequency shift observed in AuTNPs indicates effective electronic coupling between the dyes and the Au surface, thereby modulating the molecular electron density distribution. Although Ag is commonly associated with stronger intrinsic plasmonic activity, the present results show that AuTNPs provide a substantially larger enhancement factor (EF = 43.5) compared to AgTNPs (EF = 3.4). This indicates that, in this system, the overall SERS performance is governed not only by intrinsic plasmonic properties of the metal but also by factors such as spectral overlap between the excitation wavelength and the localized surface plasmon resonance (LSPR), nanoprism morphology, surface chemistry, and analyte adsorption efficiency.^32^ The significantly higher EF observed for AuTNPs suggests that the electromagnetic enhancement (EM) mechanism is considerably more efficient for the Au nanoprism architecture employed in this work. The sharp edges and anisotropic geometry of AuTNPs likely generate highly localized electromagnetic “hotspots” that better match the excitation laser and/or the molecular electronic transitions of CV and MG. Consequently, stronger near-field amplification is achieved with AuTNPs than with AgTNPs. In addition to EM enhancement, chemical enhancement also contributes to the observed spectral differences. Chemical enhancement arises from interfacial charge-transfer interactions between the adsorbed molecule and the metallic surface, and it selectively amplifies vibrational modes that are electronically coupled to the substrate.^31,35^ The changes in band positions and intensity ratios indicate that interfacial charge-transfer interactions occur on both substrates. However, the greater overall enhancement observed for AuTNPs suggests that Au provides a more favorable balance between electromagnetic amplification and molecule–surface electronic coupling in the present system. This behavior may be related to the stronger adsorption affinity of CV and MG on Au surfaces and improved stabilization of the adsorbed state, favoring more efficient plasmon-induced charge-transfer processes. Therefore, although Ag generally exhibits intrinsically stronger plasmonic behavior, the superior EF observed for AuTNPs indicates that the substrate, resonance conditions, and adsorption characteristics dominate the SERS response in this case. The spectral shifts and intensity variations support the conclusion that AuTNPs promote more effective coupling between EM and CE mechanisms, resulting in significantly higher signal enhancement for both CV and MG.^31,35,36^

The SERS spectral acquisition is suboptimal for both substrates, since their peak extinction does not coincide with the laser wavelength. Therefore, the observed differences in spectral features are most plausibly associated with the distinct compositions of the nanoprisms, which may influence analyte-surface interactions and molecular orientation at the metal interface, ultimately leading to variations in the SERS response. Another factor that could influence molecular orientation is the analyte concentration. However, average SERS spectra of CV at lower concentrations, 2 μmol L^–1^ on AgTNPs-printed and 10 nmol L^–1^ on AuTNPs-printed, corresponding to their respective lowest concentration of detection, still retain the overall spectral profile observed at the concentration studied in this work, as shown in Figure S8. This observation indicates that the composition-dependent spectral differences reported here are not primarily driven by concentration effects. Despite the AEF indicating that 785 nm excitation is suboptimal, shifting to shorter wavelengths would result in pronounced fluorescence from the functionalized paper, compromising spectral quality. An intriguing direction for future work would be to investigate substrates capable of generating third-generation hot spots, arising from the superposition of the electromagnetic fields of the metal nanoparticles with those scattered by the underlying support.^37^ Figure S9a shows spectra from the mapping of a blank substrate composed of DDSA-functionalized paper, AgTNP-printed, and AuTNP-printed regions. A blank-printed substrate is not included, as the colorless ink formulation cannot be reliably monitored during printing. Figure S9b presents the spectra obtained from nonfunctionalized chromatographic paper. Together, the blank substrates spectra in Figure S9 confirm that no major background interference arises from either the substrate or the printed inks. Figure S10 presents the Relative Standard Deviation (RSD) of the CV measurements for AgTNP-printed and AuTNP-printed substrates. The elevated RSD values likely arise from the intrinsic nonhomogeneity of the paper surface and the stochastic nature of hot-spot formation.

The different SERS responses of CV and MG on Au and Ag nanoprisms are not attributed solely to generic “surface interactions”, but rather to differences in the electronic structure of the metals and their influence on molecule–surface coupling. Au and Ag possess distinct work functions and Fermi level positions (Au ≈ 5.1 eV and Ag ≈ 4.3–4.7 eV), which directly affect interfacial charge-transfer processes between the metal surface and the frontier molecular orbitals of triphenylmethane dyes.^35,38^ Since CV and MG are highly conjugated cationic molecules with low-lying π* orbitals, the energetic alignment between the molecular LUMO and the Fermi level strongly influences adsorption strength, electron delocalization, and resonance-assisted Raman enhancement.^31,35^ Literature reports and DFT-based studies on triphenylmethane dye adsorption suggest that Ag surfaces generally promote stronger electronic coupling and greater charge-transfer contributions due to their higher-energy Fermi level and lower interband damping.^31,35,38^ This facilitates partial electron donation/back-donation between the aromatic π-system and the metal surface, producing measurable perturbations in vibrational frequencies and relative Raman intensities. Such interactions are particularly relevant for modes involving aromatic C=C stretching dimethylamino substituents, which are highly sensitive to changes in electron density distribution. In contrast, Au surfaces exhibit a more stabilized d-band electronic structure and weaker intrinsic charge-transfer activity.^31^

On the other hand, Au often provides stronger adsorption stability for aromatic cationic dyes through dispersive π-metal interactions and surface polarization effects. In our system, this appears to favor a more different coupling between the adsorbed molecules and the localized plasmonic near field, leading to the substantially higher EF observed for AuTNPs (43.5) compared to AgTNPs (3.4). Therefore, although experimental results indicate that the AuTNPs morphology produced more favorable resonance conditions and adsorption geometries for CV and MG under our excitation conditions. Additionally, crystallographic facet exposure likely contributes to the observed spectral differences. Triangular nanoprisms predominantly expose a low-index {111} facet together with highly reactive edge and tip sites. Previous theoretical and experimental studies have shown that aromatic dyes adsorb differently on Au(111) and Ag(111) surfaces due to variations in surface-atom coordination, electronic density distribution, and adsorption energy.^39,40^ Ag(111) surfaces tend to favor stronger chemisorptive interactions and more pronounced charge redistribution, whereas Au(111) surfaces often preserve greater molecular conjugation and molecular polarizability.^39–42^ These differences directly influence SERS selection rules by modifying the adsorption orientation of the triphenylmethane backbone relative to the surface. Accordingly, the distinct spectral fingerprints observed here likely arise from a combination of (i) different interfacial charge-transfer efficiencies associated with the Au and Ag electronic structures; (ii) variations in adsorption geometry governed by surface energetics and exposed facets; and (iii) differences in local electromagnetic field confinement associated with the nanoprism plasmonic response.

### 2.3 Simulations of the Electromagnetic Field Enhancement

Figure 5 shows the simulated extinction spectra of AgTNPs (a) and AuTNPs (b). The dashed curves correspond to the extinction spectra calculated for stacked dimers with the incident electric field polarized along the nanoparticle thickness direction.

**Figure 5:**
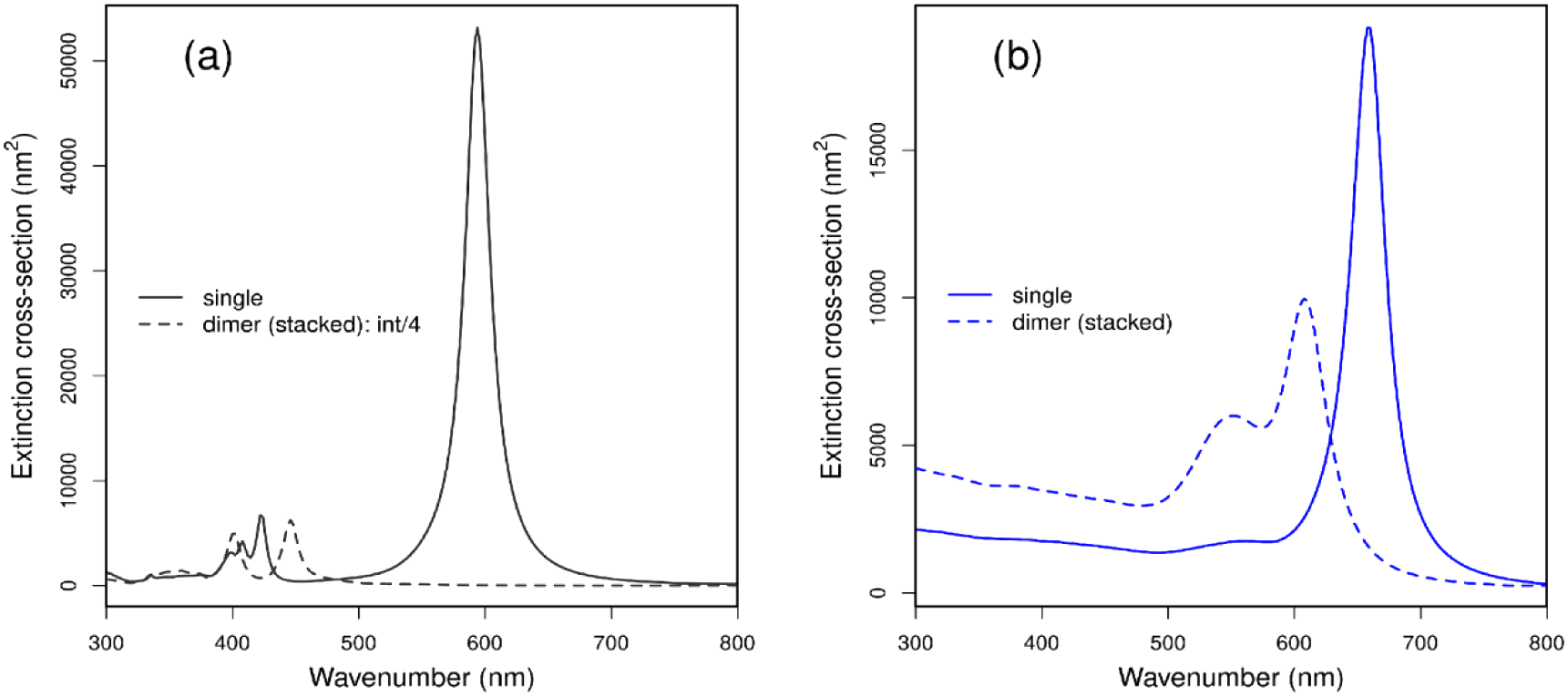
(a) Simulated extinction spectrum of a single AgTNP (solid line) compared with that of a stacked AgTNP dimer separated by a 1 nm gap (dashed line), with the excitation polarization oriented along the nanoparticle thickness direction. For direct comparison with the experimental spectra, the extinction intensity of the dimer was scaled by a factor of 4. (b) Comparison between the simulated extinction spectra of a single AuTNP (solid line) and a stacked AuTNP dimer (dashed line).

The results presented in Figure 5 are in very good agreement with the experimental data for both AgTNPs and AuTNPs. Notably, the experimental band observed around 450 nm for AgTNPs, as well as the shoulders at approximately 550 and 600 nm for AuTNPs, can be reproduced by considering stacked nanoparticle dimers in the simulations. These results suggest that this type of aggregation may already be present in the colloidal suspensions, particularly in the case of AuTNPs. During ink preparation and the subsequent printing process, nanoparticle aggregation is expected to become even more significant, leading to a variety of assembly geometries, including tip-to-tip (tt), edge-to-edge (ee), and stacked configurations. Consequently, modeling the SERS response of these systems is challenging, as multiple plasmonic coupling mechanisms may coexist within the printed material. To obtain a broader understanding of the expected SERS behavior, we performed simulations for AgTNP and AuTNP dimers in tt and ee configurations, as well as for the corresponding structures formed by stacked nanoparticles. The calculated SERS enhancement factors for 785 nm excitation are presented in Figure S10.

The field distributions calculated for ee (Figure S10a) and tt (Figure S10b) dimers composed of single nanoprisms indicate higher SERS enhancement factors for AgTNPs than for AuTNPs. However, considering the evidence of nanoprism stacking discussed above, such isolated dimer configurations are unlikely to be representative of the structures probed on the printed SERS substrates. Therefore, additional simulations were performed for ee and tt dimers formed by stacked AgTNPs and AuTNPs (Figure S10c, d). In these configurations, the plasmonic coupling between stacked nanoprisms significantly modifies the local electromagnetic fields, leading to comparable SERS enhancement factors for AgTNPs and AuTNPs when similar numbers of nanoparticles are considered. Consequently, the approximately one-order-of-magnitude higher experimental SERS intensity observed for AuTNPs cannot be explained solely by the dimer configurations investigated in Figure S10.

This discrepancy may arise from additional factors not accounted for in the present simulations, such as the formation of larger and more aggregates with more complex electromagnetic interactions and the size distribution of the nanoparticles. In particular, the microscopy images presented in the Supporting Information (Figure S3) reveal a non-negligible population of nanoparticles with edge lengths approaching 100 nm, especially in the case of AuTNPs. Since larger nanoprisms can support stronger electromagnetic enhancements at 785 nm, their contribution to the observed SERS response may be significant. To investigate this possibility, additional simulations were carried out for tt assemblies composed of AgTNPs and AuTNPs with edge lengths of 100 nm. The corresponding results are presented in Figure S11.

Larger AuTNPs arranged in ee configurations exhibit higher SERS enhancement factors than their AgTNP counterparts (see Figure S12), whereas tt configurations produce comparable enhancements for both materials. These findings indicate that the presence of larger AuTNP aggregates may contribute to the superior SERS performance observed experimentally for AuTNP-based substrates under 785 nm excitation.

### 2.4 Determination of the Molar Fraction in CV/MG Mixtures

Due to their structural similarity, the Raman/SERS spectra of crystal violet (CV) and malachite green (MG) exhibit substantial spectral overlap. Nevertheless, differences in spectral response were observed depending on the interaction between the analytes and the nanoparticle-printed substrate regions. To evaluate the possibility of predicting the molar fraction of CV/MG mixtures, an artificial neural network (ANN) model was developed using the full Raman/SERS spectra as input.

The ANN architecture, shown in Figure 6a, consisted of an input layer containing 377 spectral variables corresponding to the full Raman/SERS spectral range, followed by two hidden layers containing 16 neurons each. Prior to ANN training, the baseline-corrected spectra were normalized using the Standard Scaler procedure, which performs feature-wise mean centering and variance scaling to unit standard deviation. The ReLU activation function was used in the hidden layers, while a softmax output layer was employed to predict the relative molar fractions of CV and MG. A total of 3600 Raman/SERS spectra were used for model development, with 80% (randomly selected) of the data set employed for training and 20% reserved for validation. Model training was performed for up to 300 epochs using the Adam optimizer with default learning-rate parameters, a batch size of 32 spectra, and a mean absolute error (MAE) loss function. To reduce overfitting, dropout regularization (0.2) was applied after each hidden layer by randomly deactivating neurons during training, thereby reducing excessive dependence on specific spectral features and improving model generalization. In addition, early stopping was implemented using the validation loss as the monitored metric with a patience value of 30 epochs and restoration of the best model weights, preventing unnecessary training after convergence. The corresponding training and validation learning curves are presented in Figure 6b and demonstrate stable convergence behavior throughout the optimization process.

**Figure 6:**
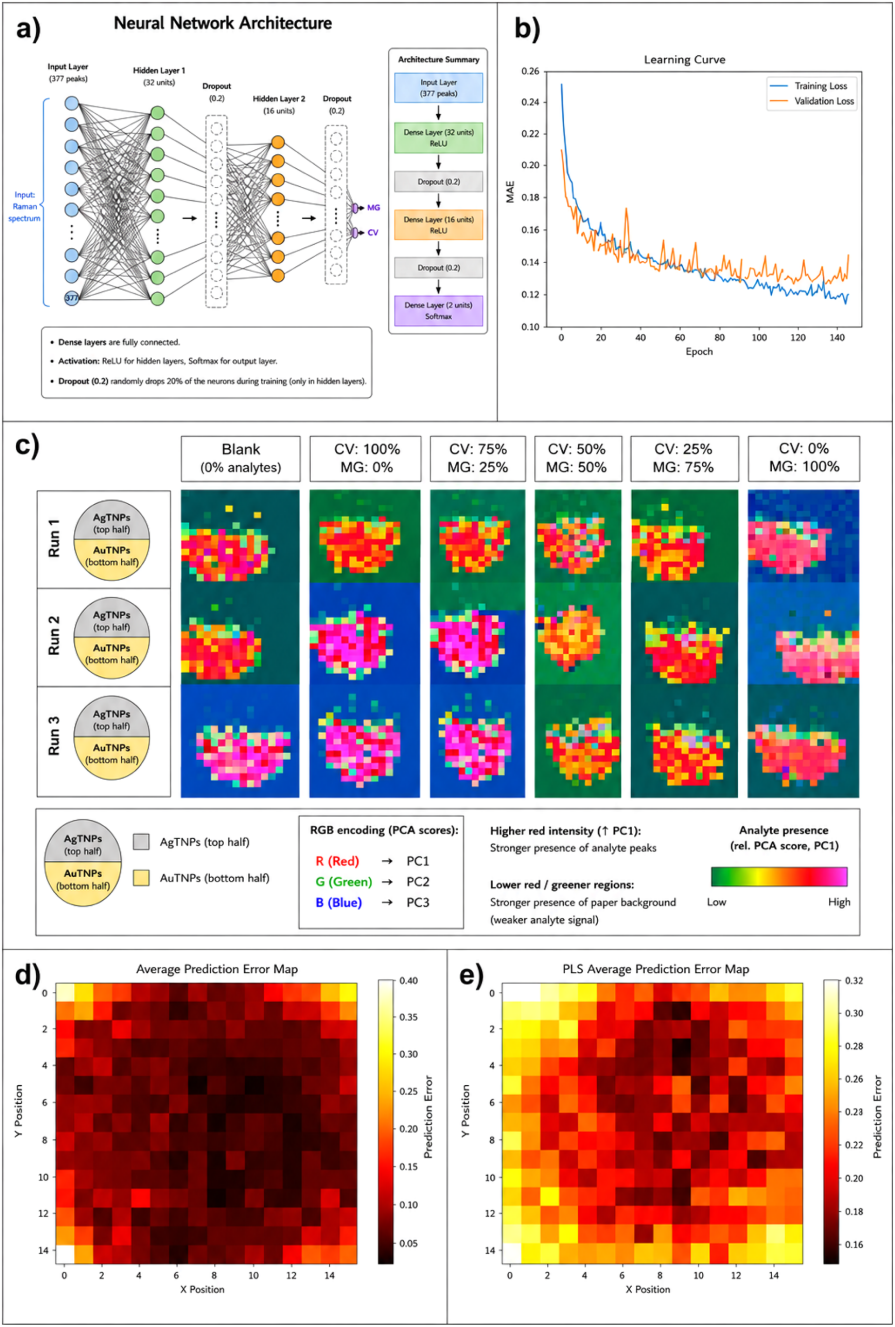
(a) ANN architecture. (b) Training and validation learning curves. (c) PCA-based reconstructed Raman/SERS mappings for different CV/MG molar fractions. (d) ANN MAE heat maps for molar-fraction prediction. (e) Corresponding PLS MAE heat maps.

Prior to principal component analysis (PCA) and ANN training, all spectra were baseline-corrected using polynomial fitting. The spectra were subsequently mean-centered and normalized using L2 vector normalization. No additional variance scaling was applied. PCA was subsequently performed on the normalized spectra to evaluate spectral variance across the mapped substrate regions and to reconstruct the spatial Raman/SERS mappings presented in Figure 6c. The ANN model produced MAE, RMSE, and R^2^ values of 0.080, 0.147, and 0.828, respectively, for the training data set, and 0.106, 0.174, and 0.757 for the validation data set. Although the validation error exceeded the training error, the learning curves indicate that the use of dropout regularization and early stopping reduced excessive model memorization while preserving predictive capability. To further evaluate model performance, partial least-squares (PLS) regression was also applied as a baseline method for SERS quantification. The spatial prediction-error heat maps obtained for the ANN and PLS models are shown in Figure 6d, e, respectively. Under the present experimental conditions, the ANN model produced lower spatial prediction errors than the PLS approach, particularly in regions exhibiting stronger analyte-related Raman contributions.

The reconstructed Raman/SERS mapping images shown in Figure 6c were reconstructed from PCA-transformed Raman/SERS spectra obtained at each spatial coordinate of the mapped substrate. Each pixel therefore corresponds to one Raman/SERS spectrum collected from a specific position on the nanoparticle-printed paper substrate. The RGB (Red, Green, Blue) channels were assigned according to the normalized PCA scores (R = PC1, G = PC2 and B = PC3). In particular, higher red-channel intensity, associated with larger PC1 scores, corresponds to spectra exhibiting stronger analyte-related Raman contributions, whereas greener regions are predominantly associated with spectral contributions from the paper substrate/background. The upper half of each reconstructed mapping corresponds to AgTNP-printed regions, whereas the lower half corresponds to AuTNP-printed regions, as indicated in Figure 6c.

The reconstructed mappings reveal that regions dominated by paper-background contributions exhibit lower analyte-associated spectral intensity and higher prediction uncertainty. This behavior is particularly evident near the edges of the printed regions and in AgTNP-printed areas, which exhibited comparatively weaker SERS enhancement. The spatial MAE heat maps shown in Figure 6d, e further demonstrate that the largest prediction errors are localized predominantly in substrate-dominated regions, where the spectral contrast between CV, MG, and the paper substrate becomes insufficient for robust discrimination.

## 3 Conclusion

The experimental results obtained in this study are likely influenced by a combination of factors related to the nanoparticles’ composition and morphology. Although AuTNPs and AgTNPs exhibit comparable overall geometries, differences in size distribution and morphology purity coupled with the possibility that each nanoprism composition might expose slightly different proportions of crystallographic facets, contributes to the observed outcomes. However, it is unlikely that surface saturation of the nanoparticles due to high molecular concentrations plays a significant role, as the characteristic spectral profiles are preserved even at lower analyte concentrations. Nonetheless, it would be valuable to disentangle the effects of nanoparticle composition from other confounding factors present in the current substrate. Nanoparticle concentration and substrate surface saturation could be systematically evaluated by varying either the number of print passes or the ink concentration. The influence of the substrate itself could be further examined by employing fabrication methods compatible with rigid substrates. Such studies would help evaluate the contribution of each parameter and yield a deeper understanding of the relationship between nanoparticle composition and the resulting SERS response.

The combined analysis of SERS spectra acquired from analytes interacting with different plasmonic nanostructures enables both the discrimination and quantification of closely related molecules when supported by appropriate multivariate tools. The present results further indicate that effective implementation of this strategy requires plasmonic components with sufficiently high and comparable enhancement factors, particularly at low analyte concentrations. Optical stability of the printed substrates should also not be disregarded, particularly for AgTNPs-printed, as it limits their practical applicability. To further elucidate the extent of silver oxidation, XPS analysis of aged AgTNP-printed substrates will be pursued in future work. This issue could potentially be mitigated by adopting a print-and-use strategy, given that the nanoparticle inks themselves exhibit much longer stability. Finally, inkjet printing of nanoparticle inks onto paper substrates provides a reproducible and versatile approach for integrating multiple plasmonic nanostructures within a single analytical platform, offering a robust framework for systematically investigating composition-dependent SERS responses.

## 4 Materials and Methods

### 4.1 Reagents and Solutions

For the synthesis of silver triangular nanoprisms (AgTNPs), sodium borohydride (≥99%), trisodium citrate dihydrate (≥98%), and silver nitrate (≥99%) were purchased from Sigma-Aldrich, while sodium hydroxide (97%) was obtained from Synth. For the synthesis of gold triangular nanoprisms (AuTNPs), hydrogen tetrachloroaurate­(III) trihydrate (HAuCl_4_·3H_2_O, ≥99.9%), cetyltrimethylammonium chloride (CTAC, 25 wt % aqueous solution), and L-ascorbic acid (≥99%) were purchased from Sigma-Aldrich; sodium iodide (99.5%) was obtained from Merck, and sodium hydroxide (97%) from Synth. For paper surface modification, cellulose chromatography paper (Whatman), (2-dodecen-1-yl)­succinic anhydride (DDSA, 95%), and 1-hexanol (98%) were obtained from Sigma-Aldrich. For the preparation of nanoprism inks, glycerol (≥99.5%) and absolute ethanol (99.5%) were purchased from Sigma-Aldrich and Anidrol, respectively. Crystal violet (CV, ≥90%) was obtained from Sigma-Aldrich, and malachite green (MG, ≥90%) from Synth.

All glassware was cleaned with freshly prepared aqua regia (3:1 v/v HCl/HNO_3_) and thoroughly rinsed with deionized water prior to use. All solutions were prepared using ultrapure water (resistivity ≥ 18.2 MΩ·cm) obtained from a Milli-Q water purification system (Millipore).

### 4.2 Synthesis of Au and Ag Triangular Nanoprisms

Synthesis of AgTNPs. Silver triangular nanoprisms (AgTNPs) were synthesized via a plasmon-mediated route following the method reported by Xue et al.^43^ In a typical procedure, 24.25 mL of deionized water was mixed with aqueous solutions of silver nitrate (250 μL, 10 mmol·L^–1^) and sodium citrate (250 μL, 100 mmol·L^–1^) at room temperature. Subsequently, 250 μL of an aqueous mixture containing sodium borohydride (8 mmol·L^–1^) and sodium hydroxide (0.125 mol·L^–1^) was added dropwise in the course of 10 s under continuous stirring (600 rpm). Immediately after reagent addition, the reaction mixture was irradiated with a 70 W sodium lamp (Osram) for 2 h while stirring, in a distance around 5 cm from the glassware. The resulting AgTNP suspensions were stored protected from light and kept under refrigeration until further use. Synthesis of AuTNPs. Gold triangular nanoprisms (AuTNPs) were synthesized using the seedless method described by Kim et al.^44^ Briefly, the following aqueous solutions were sequentially added to 8.0 mL of deionized water at room temperature in the following order: cetyltrimethylammonium chloride (CTAC, 1.6 mL, 100 mmol·L^–1^), chloroauric acid (80 μL, 25.4 mM), sodium iodide (75 μL, 10 mM), sodium hydroxide (20 μL, 100 mM), ascorbic acid (80 μL, 64 mM), and finally sodium hydroxide (10 μL, 100 mM). Gentle manual stirring was applied only to homogenize the solution after each addition. The reaction mixture underwent a characteristic color change from red to blue, and after standing for 10 min at room temperature, the synthesis was considered complete. Illustrative schematics of both synthetic procedures are provided in the Supporting Information, Figure S1a, b.

### 4.3 Production of Inkjet-Printed SERS Substrates

Ink preparation. The AuTNP and AgTNP colloids were concentrated by two consecutive centrifugation steps. Initially, the colloids were centrifuged for 10 min at 10,000 rpm for AuTNPs and 6500 rpm for AgTNPs. A second centrifugation step was then performed for both colloids at 10 000 rpm for an additional 10 min. After each step, the supernatant was discarded to adjust the metal concentration in the colloids to approximately 20 mmol L^–1^. The concentrated colloids were subsequently mixed with glycerol and ethanol in a volumetric ratio of 65:25:10 (colloid:glycerol:ethanol) to obtain inks with viscosities comparable to those of commercial inkjet formulations.^14^ Paper surface modification. Chromatography paper was rendered hydrophobic following the procedure optimized by Godoy et al.^14^ Briefly, the paper was immersed in a 0.4% (w/v) solution of (2-dodecen-1-yl)­succinic anhydride (DDSA) in 1-hexanol for 5 min, followed by thermal curing in an oven at 160 °C for at least 6 min. This treatment cycle was repeated three times to ensure uniform surface modification. Printing substrates. The AuTNP and AgTNP inks were printed onto the functionalized paper using a commercial inkjet printer (Epson L3250). Circular SERS substrates with a diameter of approximately 2 mm were fabricated using a side-by-side design, in which each ink occupied one semicircle of the printed area. Five overlapping print passes were applied to increase nanoparticle loading and signal reproducibility. Prints underwent under room temperature and humidity. All printing steps were performed using the highest quality setting available in the printer software, 300 dpi (dots per inch).

### 4.4 Substrate Characterization and SERS/Raman Measurements

VIS-NIR extinction spectra of the inks were recorded using a Cary 8453 spectrophotometer (Agilent). Diffuse reflectance VIS-NIR extinction spectra of the inkjet-printed SERS substrates were recorded using a Shimadzu UV-2450 spectrophotometer. Zeta potential measurements were performed using Malvern (ZS-Zen 3600) Particle Size and Zeta Potential Analyzer. Scanning electron microscopy (SEM) images of the nanoprisms and printed substrates were acquired using a JEOL JSM-6700F field-emission scanning electron microscope operated at an accelerating voltage of 10.0 kV in backscattered electron mode (YAG detector). Prior to SEM analysis, substrate samples were sputter-coated with a thin platinum layer to improve surface conductivity. Nanoparticle dimensions were quantified from SEM micrographs using ImageJ (NIH). Images were first calibrated using the scale bar to convert pixel measurements into nanometers. Particles were manually measured. At least 200 nanoparticles per sample were measured to obtain representative size distributions. Raman and SERS spectra were collected using a HORIBA Scientific XploRA ONE Raman spectrometer coupled to a Jobin Yvon confocal microscope and a charge-coupled device (CCD) detector. Wavenumber calibration was performed using the first-order silicon band at 520.2 cm^–1^. For SERS measurements, 2 μL aliquots of aqueous solutions were drop-cast onto the printed areas of the substrates. These solutions contained crystal violet (CV) and malachite green (MG) either individually or as binary mixtures at molar fractions of 25%, 50%, and 75%, maintaining a total dye concentration of 10 μmol L^–1^. After deposition, the samples were allowed to dry at ambient conditions prior to spectral acquisition. Measurements were performed over a three-day period following printing, with the substrates stored in a desiccator whenever they were not being analyzed. Spectra were recorded using a 785 nm excitation laser, with an acquisition time of 15 s, one accumulation, 50% laser power, and a ×10 objective lens. All Raman and SERS spectra were background-subtracted using the instrument software (HORIBA), applying a sixth-order polynomial baseline correction. Data processing and multivariate analysis were performed using Origin software and custom Python scripts for principal component analysis (PCA) and artificial neural network (ANN) modeling.

### 4.5 Computational Simulations

Electromagnetic simulations were performed using the boundary element method (BEM) implemented in the MNPBEM17 package.^45,46^ The optical properties of gold and silver were described using the experimental dielectric functions reported by Johnson and Christy.^47^ For simulations of the colloidal systems, water was considered as the surrounding dielectric medium, whereas simulations of nanoparticles deposited on paper were carried out assuming air as the external medium. Unless otherwise stated, the incident electric field polarization was oriented along the height of the triangular nanoprisms. The edge lengths of AgTNPs and AuTNPs were set to 70 and 55 nm, respectively, although simulations with nanoparticles of edge lengths of 100 nm were also considered. Since the nanoparticle thicknesses could not be directly determined from the microscopy images, approximate values were estimated through direct comparison between the simulated and experimental extinction spectra, resulting in thicknesses of 15 nm for AgTNPs and 20 nm for AuTNPs. The nanoprisms were generated by 3D extrusion of triangular meshes, with the vertices rounded using circular arcs of radius r (1 nm for AuTNPs and 6 nm for AgTNPs) to reproduce the rounded morphologies observed in the microscopy images. SERS enhancement factors were calculated using E4-approximation for excitations at 785 nm.^48,49^

## Supplementary Material



## Data Availability

All data supporting the findings of this study are available within the article and its Supporting Information.

## Notes

Figure S1: schematic description of the overall synthesis procedure; Figures S2–S3: additional SEM images of nanoparticles; Figure S4: SEM micrographs of printed nanoparticles on functionalized paper; Figure S5: normalized extinction spectra of nanoparticle inks and printed substrates over time; Figures S6–S7: SERS spectra for each proportion of the mixture of CV and MG; Figure S8: average SERS spectra of CV at its respective lowest concentration tested for each substrate; Figure S9: spectra of blank substrates; Figure S10: relative standard deviation of CV for AgTNP-printed and AuTNP-printed substrates; Figure S11: simulated SERS enhancement factors for nanoprism configurations; Figure S12: simulated SERS enhancement factors for 100 nm edge nanoprisms; Figure S13: simulated spatial distributions of the SERS enhancement factors for 100 nm edge nanoprisms (PDF)

## Notes

L.M.F.O.: Conceptualization, Original draft preparation, Data curation, Validation, Writing – review and editing. A.D.B.: Conceptualization, Discussion of results, Writing – review and editing, Cosupervisor. E.H.-M.: Conceptualization, Review and editing. G.C.P.: Formal analysis, Review and editing. D.P.D.S.: Formal analysis, Review and editing. F.A.S.: Conceptualization, Review and editing, Funding acquisition. I.O.M.: Supervisor, Conceptualization, Writing – review and editing, Funding acquisition
