# Supplementary material for: Composition-Dependent SERS Responses of Structurally Related Dyes on Inkjet-Printed Paper Substrates with Gold and Silver Triangular Nanoprisms
Source: ACS Omega. 2026 Jul 20;11(30):45270–82. doi: 10.1021/acsomega.6c04353 (PMC13448946; doi:10.1021/acsomega.6c04353)
Supplement: Supplementary file 1 [file ao6c04353_si_001.pdf]

Supplementary Material

Composition-Dependent SERS Responses of Structurally Related Dyes on Inkjet-Printed Paper Substrates with Gold and Silver Triangular Nanoprisms

Lucas M. F. Oliveira[1], Anerise de Barros[1], Edison Huertas-Montoya[1], Gabriel C. Pereira[1], Diego P. dos Santos[1], Fernando A. Sigoli[1], Italo O. Mazali[1]*

[1] Universidade Estadual de Campinas, Instituto de Química, Laboratório de Materiais Funcionais, Campinas, SP, Brasil.

*Corresponding author: mazali@unicamp.br

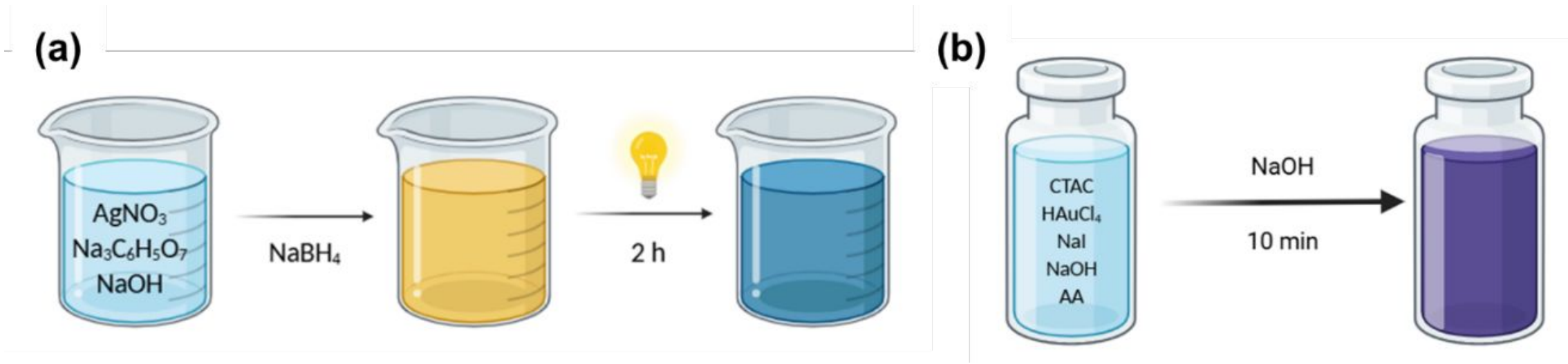

Figure S1 – Schematic description of the overall synthesis procedure of (a) AgTNPs and (b) AuTNPs. CTAC: Cetyltrimethylammonium Chloride. AA: Ascorbic acid. Created using BioRender.com.

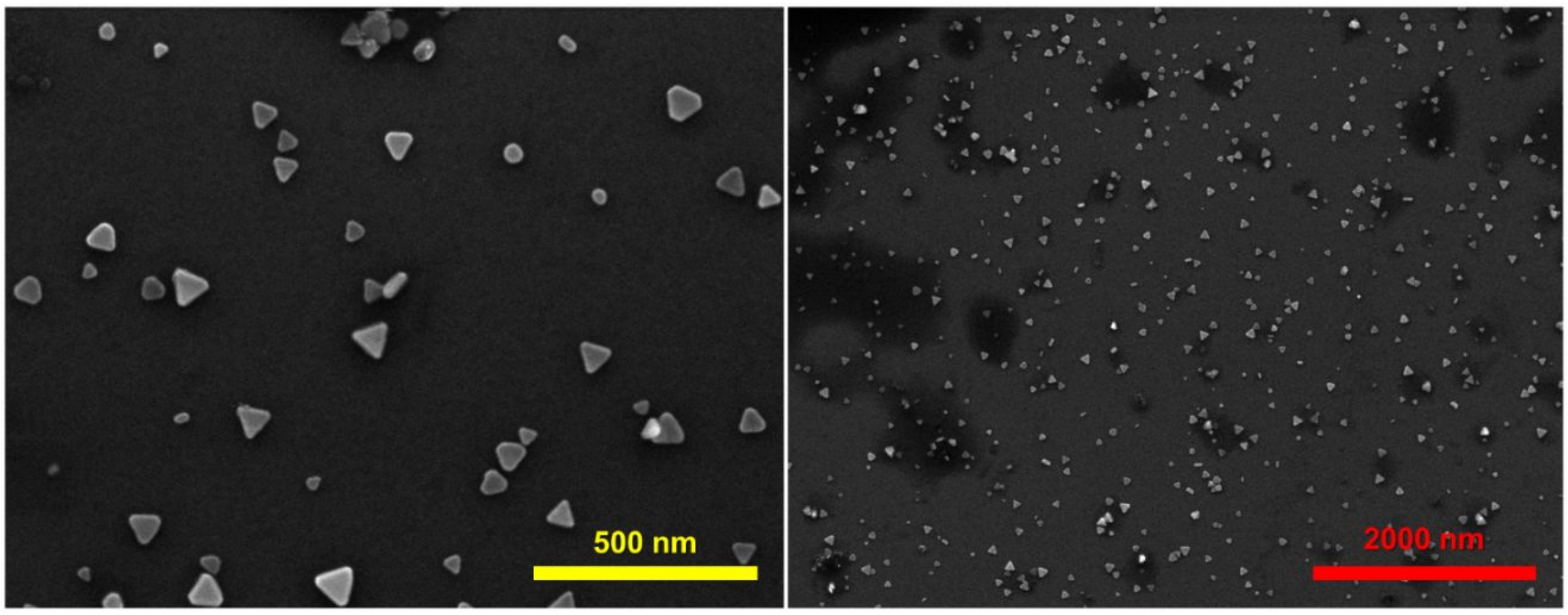

Figure S2 – Additional SEM images of AgTNPs.

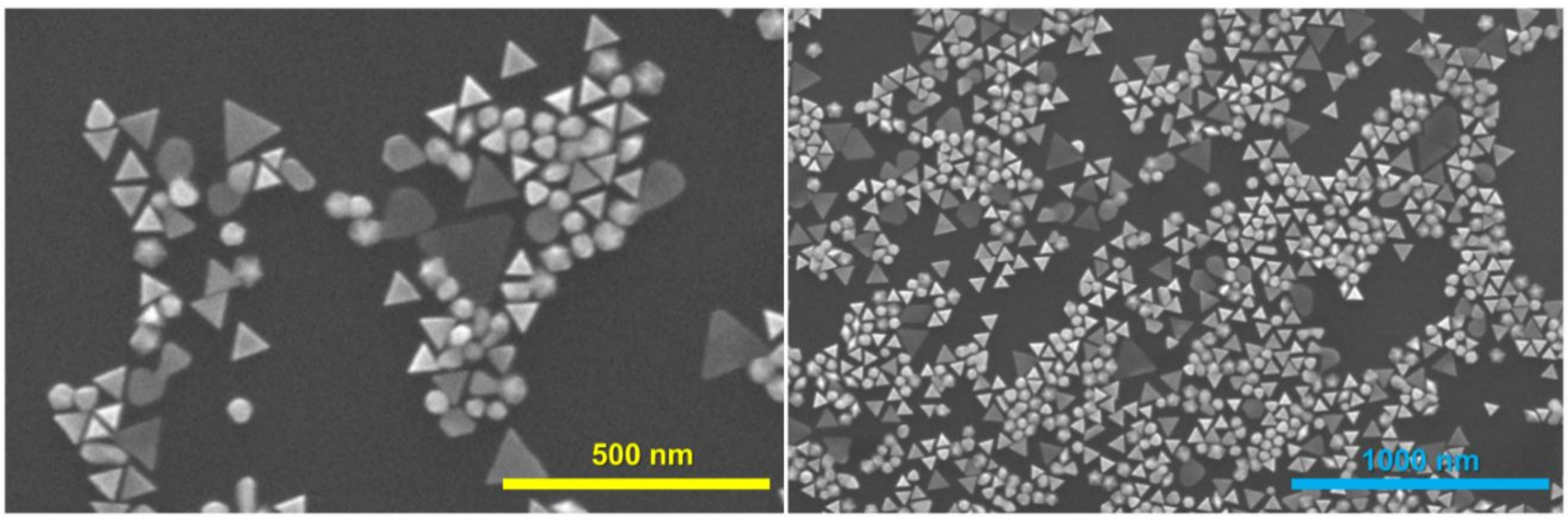

Figure S3 – Additional SEM micrographs of AuTNPs.

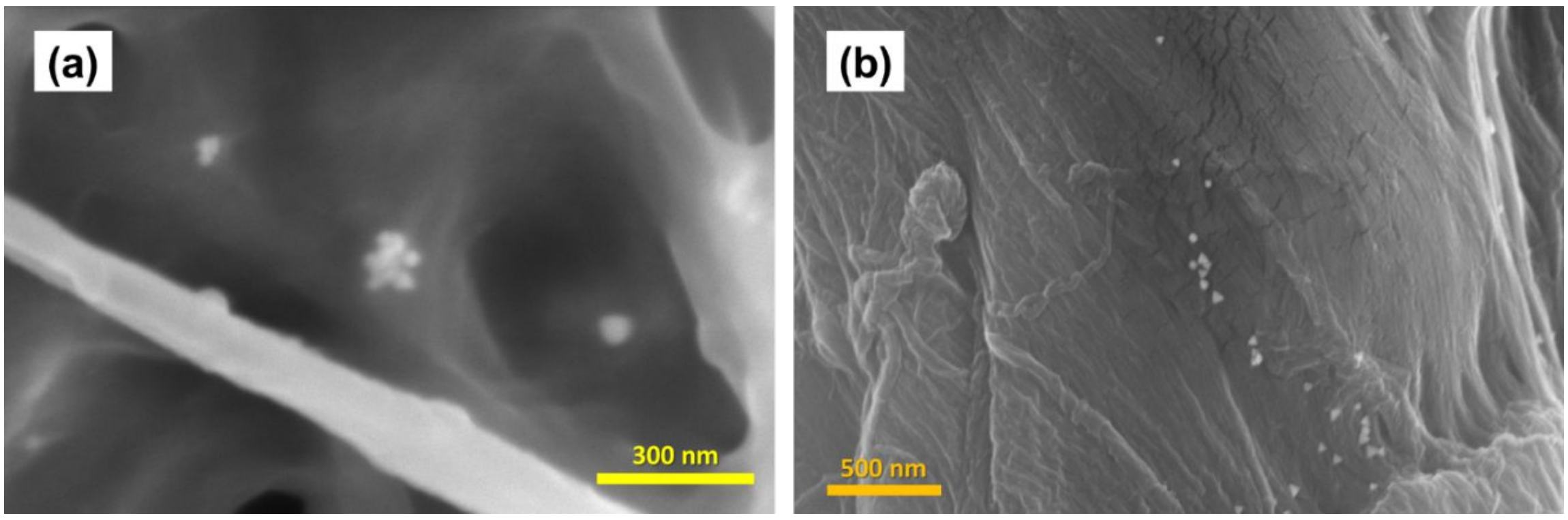

Figure S4 – SEM micrographs of (a) AgTNPs and (b) AuTNPs printed on paper. Each solely printed on different substrates.

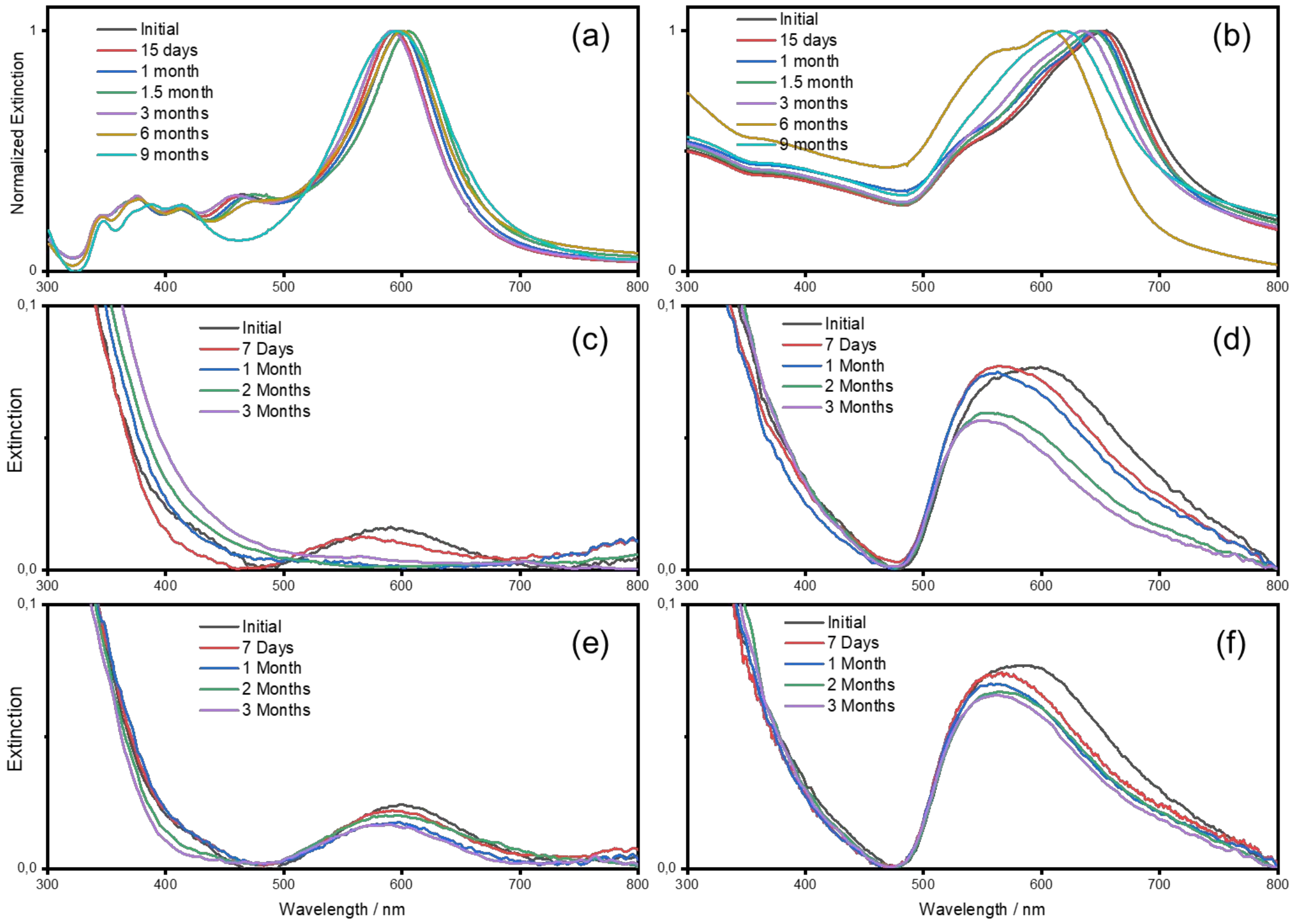

Figure S5 – Normalized extinction spectra of (a) AgTNPs and (b) AuTNPs inks over time. Normalization to the most intense band. Base line corrected extinction spectrum of printed substrates stored under atmospheric oxygen exposure, (c) AgTNPs and (d) AuTNPs, and in desiccator, (e) AgTNPs and (f) AuTNPs.

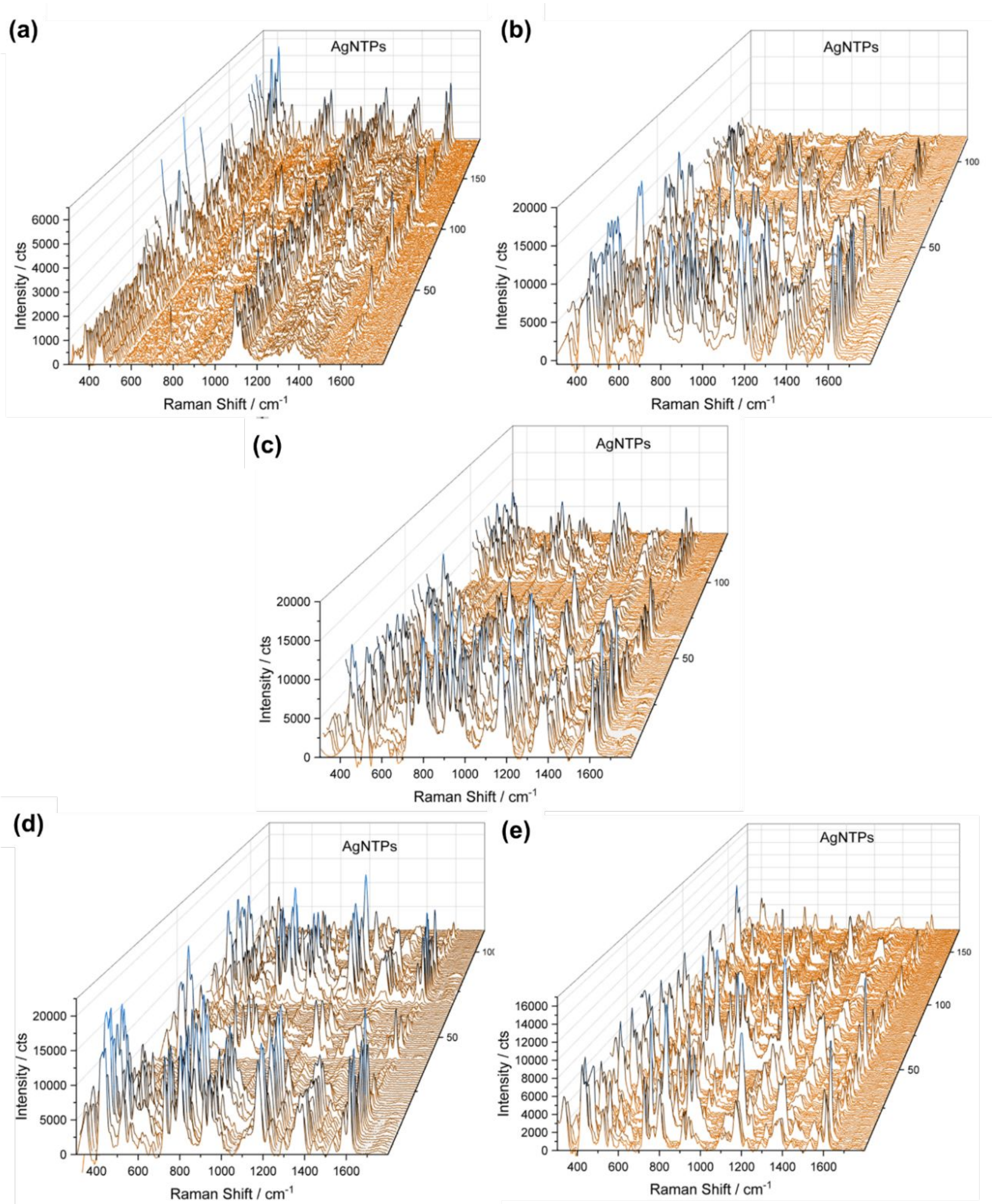

Figure S6 – SERS spectra for each proportion of the mixture CV and MG on AgNTPs. (a) CV: 0%, MG: 100%; (b) CV: 25%, MG: 75%; (c) CV: 50%, MG: 50%; (d) CV: 75%, MG: 25%; (e) CV: 100%, MG: 0%.

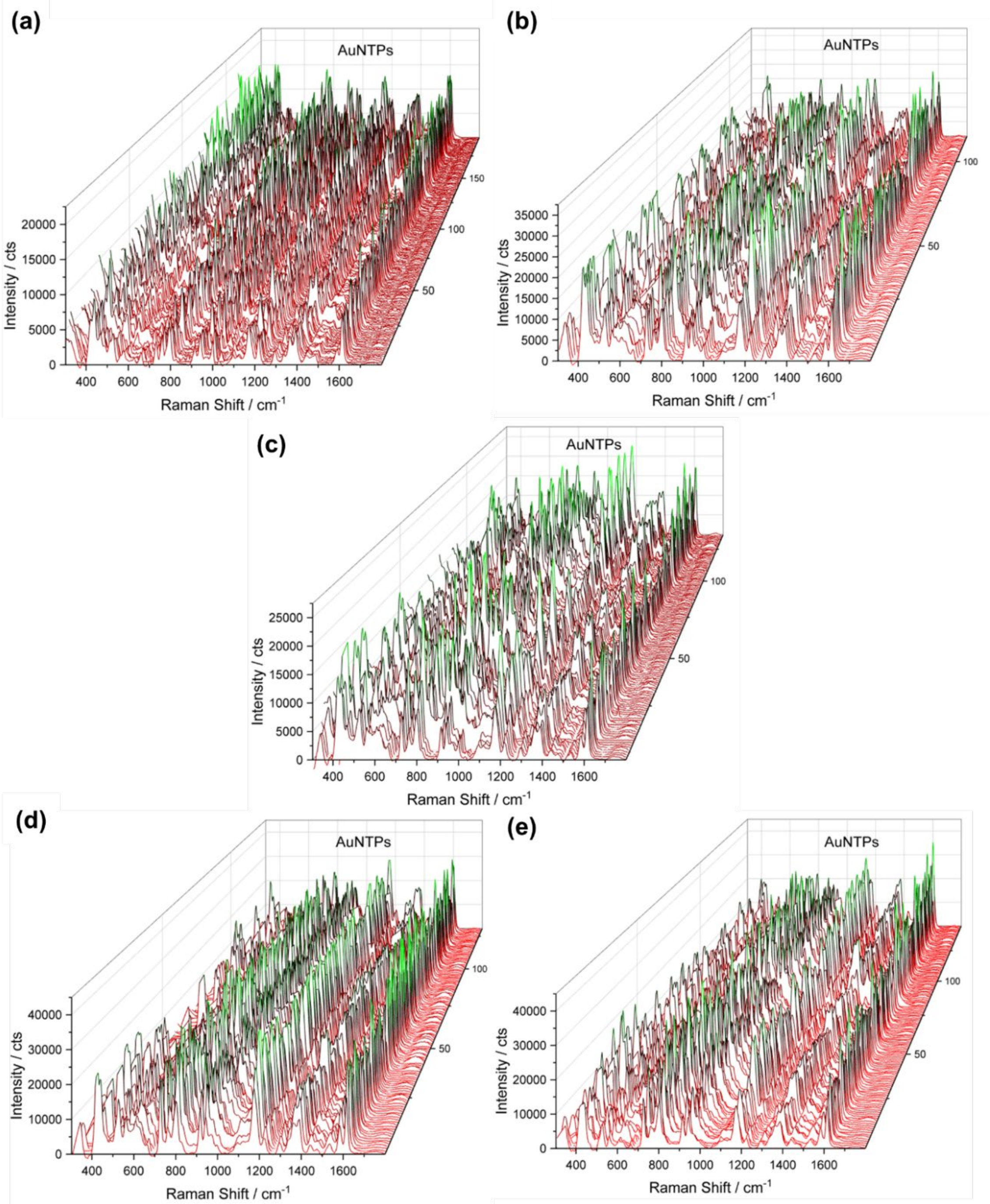

Figure S7 – SERS spectra for each proportion of the mixture CV and MG on AuNTPs. (a) CV: 0%, MG: 100%; (b) CV: 25%, MG: 75%; (c) CV: 50%, MG: 50%; (d) CV: 75%, MG: 25%; (e) CV: 100%, MG: 0%.

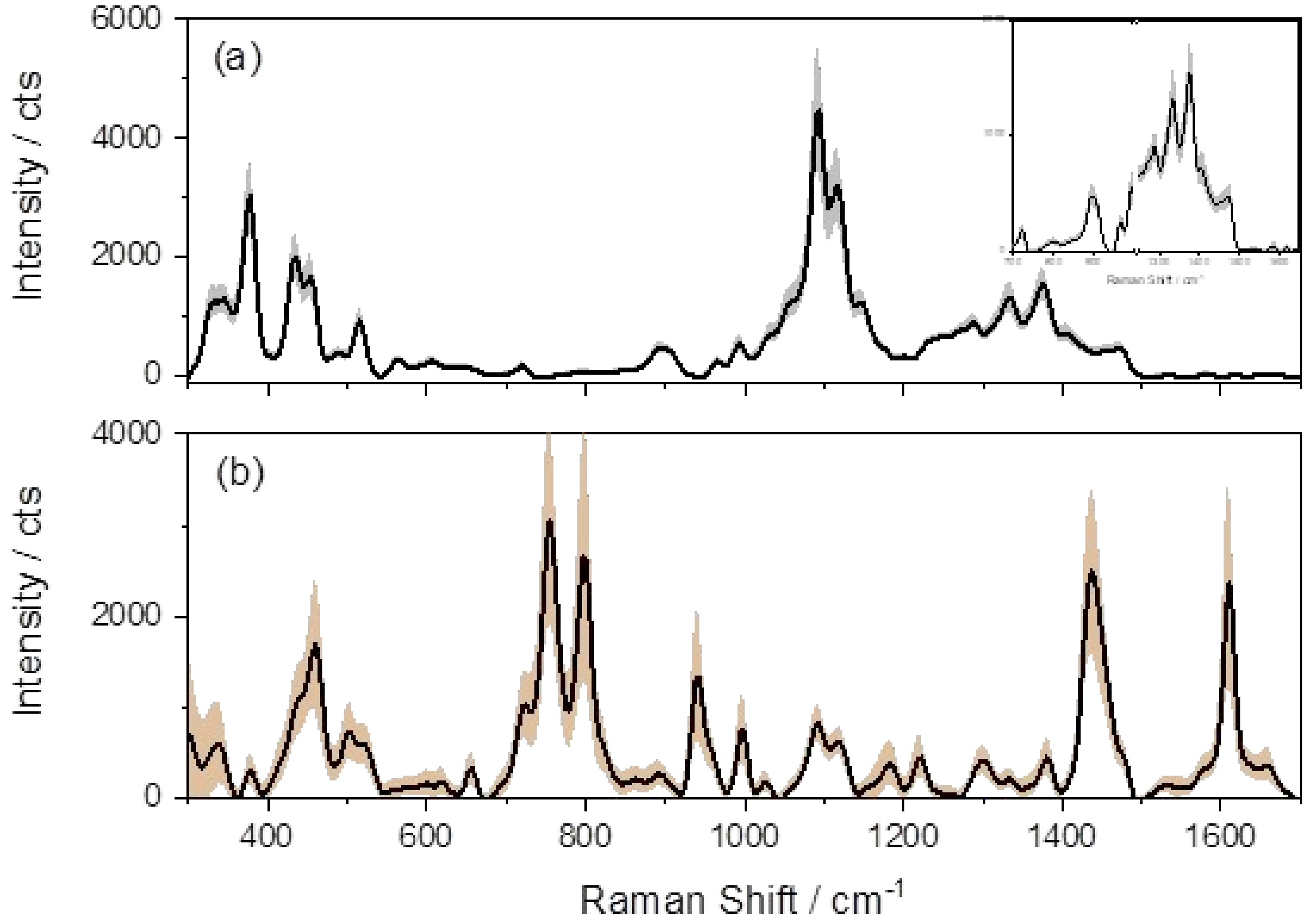

Figure S8 – Average spectra of CV interacting with (a) AgTNPs and (b) AuTNPs, at their respective lowest concentration tested, 2 μmol L^{-1} and 10 nmol L^{-1}. Shaded areas correspond to the respective standard deviations. The analysis using AgTNPs was conducted at a power density of 50% to achieve detection at this concentration.

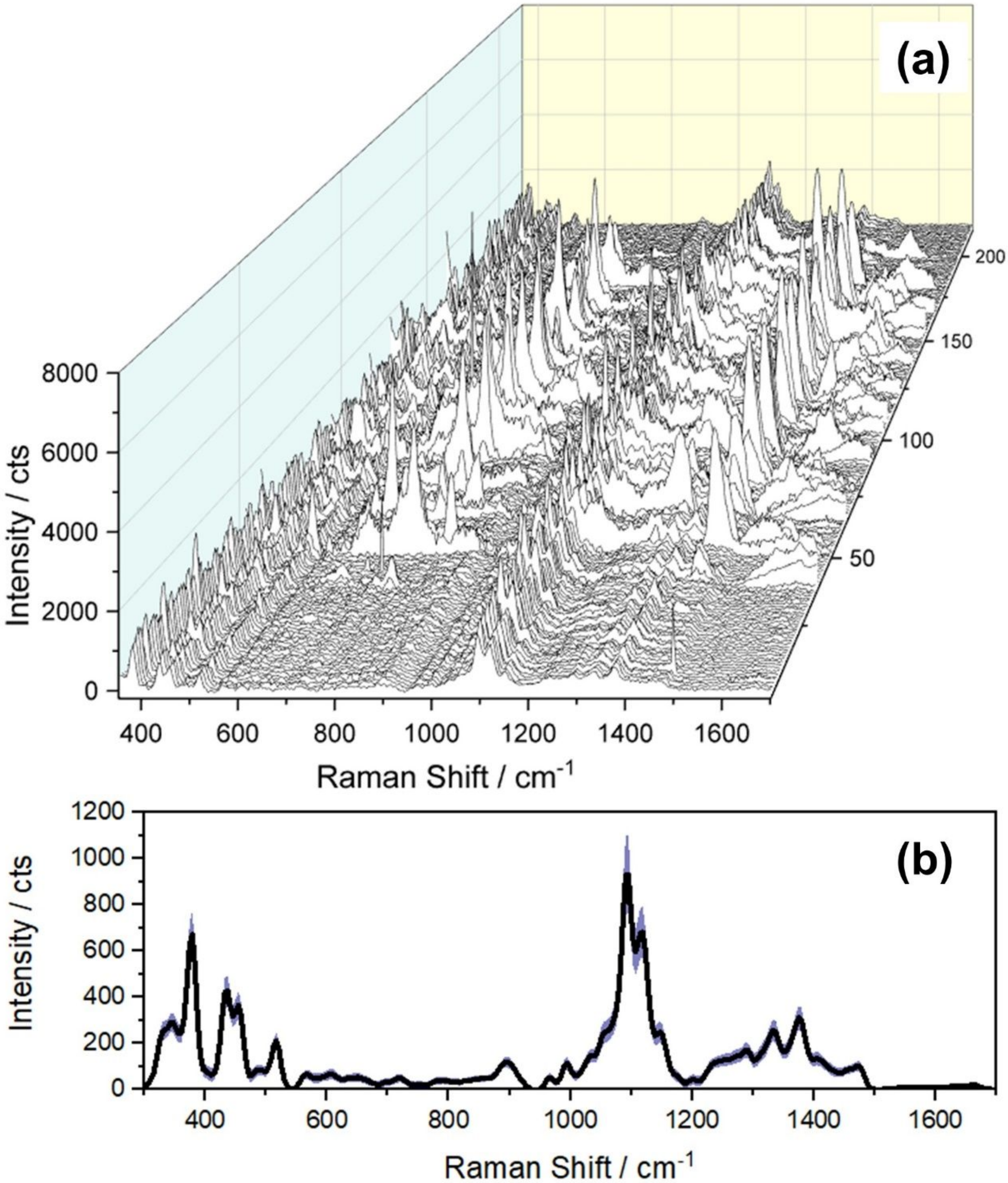

Figure S9 – (a) SERS spectra for a substrate in which was not drop casted a solution of the analytes, a blank substrate. (b) Average spectra of an unmodified chromatography paper. Shaded areas correspond to the respective standard deviations.

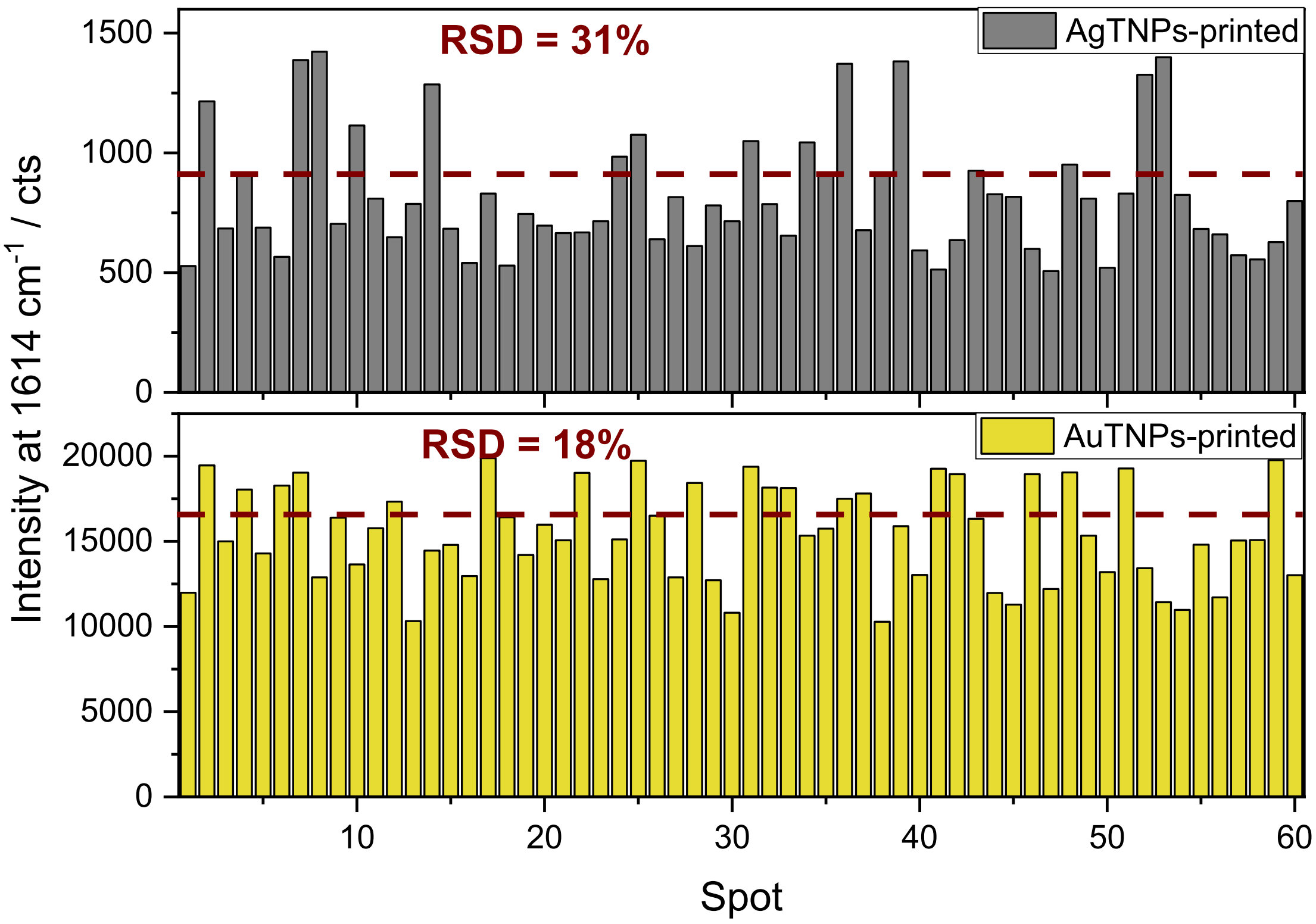

Figure S10 – Relative Standard Deviation (RSD) of CV for (a) AgTNP-printed and (b) AuTNP-printed substrates, calculated across three substrates with 20 spots per substrate.

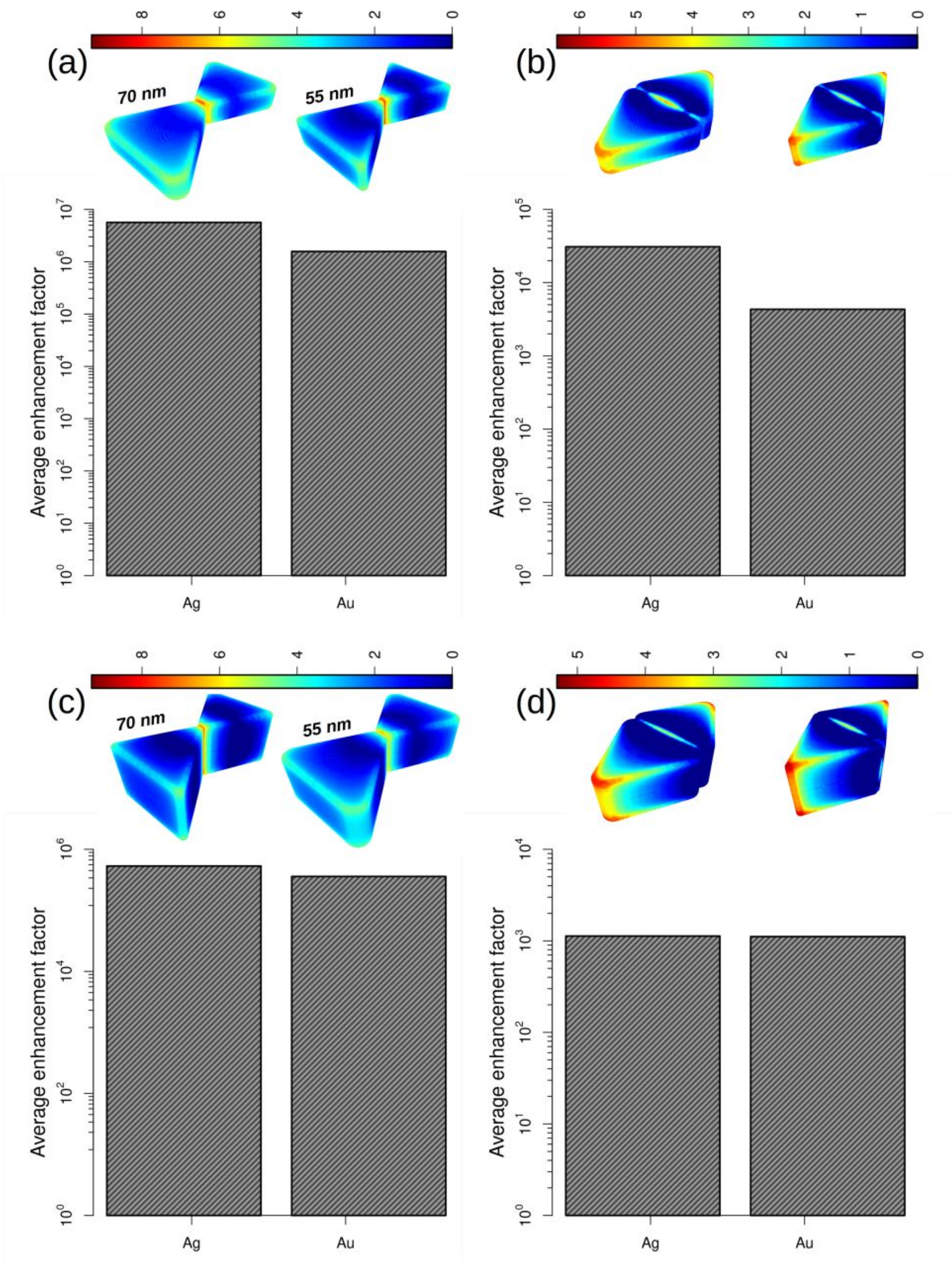

Figure S11 – (a,b) Simulated SERS enhancement factors for non-stacked AgTNP and AuTNP dimers in tip-to-tip and edge-to-edge configurations. (c,d) Simulated SERS enhancement factors for stacked AgTNP and AuTNP dimers in the same configurations. The colored maps represent the spatial distribution of the enhancement factors over the nanoparticle surfaces (logarithmic scale), whereas the bar plots show the surface-averaged enhancement factors.

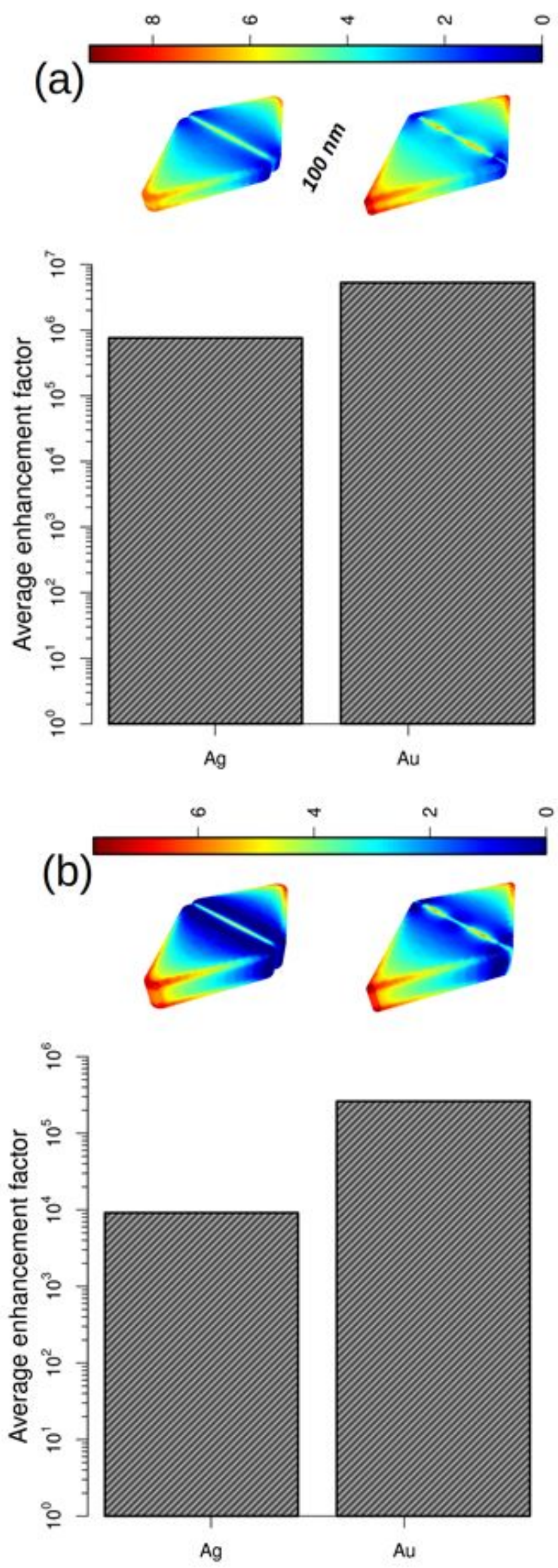

Figure S12 – Simulated SERS enhancement factors for edge-to-edge dimers composed of 100 nm AgTNPs and AuTNPs. Results are shown for (a) single nanoprisms and (b) stacked nanoprisms. The colored maps represent the spatial distribution of the enhancement factors over the nanoparticle surfaces (logarithmic scale), whereas the bar plots show the surface-averaged enhancement factors.

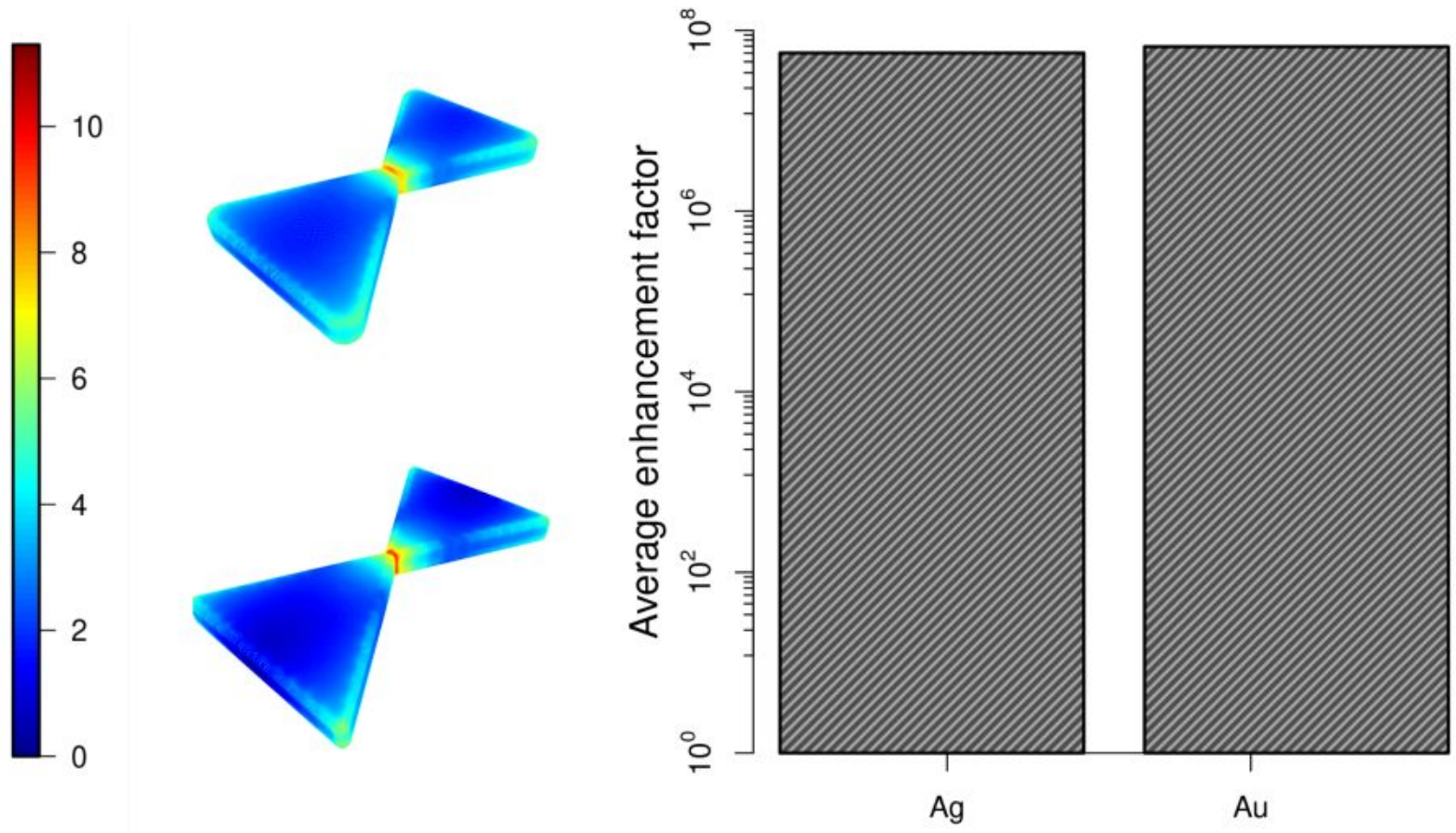

Figure S13 – Simulated spatial distributions of the SERS enhancement factors (colored maps) for edge-to-edge (ee) dimers composed of 100 nm AgTNPs (top) and AuTNPs (bottom). The bar plots represent the corresponding surface-averaged enhancement factors.
